# Epidemiological investigation of poultry infectious in Kazakhstan (2021–2024)

**DOI:** 10.3389/fvets.2024.1520606

**Published:** 2025-02-11

**Authors:** Karlygash B. Zikibayeva, Asset A. Svanbayev, Nurlan N. Akhmetsadykov, Kamshat N. Kudaibergenova, Shynar N. Akhmetsadykova, Ernur N. Nurolda, Aidyn I. Kydyrmanov

**Affiliations:** ^1^Kazakh National Agrarian Research University, Almaty, Kazakhstan; ^2^Al-Farabi Kazakh National University, Almaty, Kazakhstan; ^3^Limited Liability Partnership "Antigen", Almaty, Kazakhstan; ^4^Research and Production Center for Microbiology and Virology, Almaty, Kazakhstan

**Keywords:** poultry infectious diseases, Kazakhstan, avian influenza, newcastle disease, mycoplasmosis, infectious bursal disease, chicken anemia virus, epidemiology

## Abstract

**Introduction:**

This study examines the epidemiological dynamics and genetic diversity of major avian infectious diseases in Kazakhstan, including highly pathogenic avian influenza (HPAI), Newcastle disease virus (NDV), and others. Using official data, laboratory diagnostics, and surveys, we identified high prevalence rates and virulent strains, exposing gaps in vaccination coverage and biosecurity practices. Continuous monitoring, improved vaccination strategies, and robust biosecurity measures are essential to reduce disease impact and ensure sustainable poultry farming.

**Methods:**

A cross-sectional study was conducted to assess the prevalence and genetic diversity of major avian infectious diseases in Kazakhstan. Data sources included official reports, laboratory diagnostics (RT-PCR, ELISA, and sequencing), and a survey of veterinary specialists. Serum samples were analyzed to evaluate antibody responses and vaccine efficacy. Genetic and phylogenetic analyses were conducted for key pathogens, while a questionnaire provided insights into farm-level disease control practices.

**Results:**

Analysis of official data recorded 27 outbreaks of avian diseases in Kazakhstan from 2005 to 2023, primarily involving HPAI and NDV. Our research further identified virulent strains such as NDV genotype VII and infectious bursal disease virus (IBDV) variants linked to global lineages. Serological studies revealed widespread exposure to pathogens, including Mycoplasma gallisepticum (MG), Mycoplasma synoviae (MS), chicken anemia virus (CAV), Ornithobacterium rhinotracheale (ORT), and low-pathogenic avian influenza (LPAI) H9, underscoring deficiencies in vaccination coverage. Farm surveys also identified weaknesses in biosecurity measures and inconsistencies in vaccination protocols.

**Discussion:**

The findings underscore the urgent need for enhanced biosecurity measures, standardized vaccination programs, and routine monitoring to mitigate the impact of avian infectious diseases. This integrated approach offers valuable insights to support evidence-based decision-making for effective poultry health management in Kazakhstan.

## 1 Introduction

Kazakhstan’s poultry industry is at a critical point of significant growth but faces challenges that could impact its future. Global demand for poultry is increasing due to its affordability and health benefits. Therefore, ensuring the health of poultry flocks is crucial. To meet consumer expectations and ensure food safety, it’s essential to stay updated on animal health and disease prevention (1). Poultry meat is expected to be a major source of protein globally, accounting for 41% of all meat protein by 2030 (2).

Global chicken meat production is currently dominated by America and Asia (3). In Central Asia, a comparative analysis of the poultry populations by Abidov et al. (4) revealed that Uzbekistan leads with approximately 97 million poultry, while Kazakhstan ranks second with around 50 million, and Kyrgyzstan has a much smaller population of about 6 million.

While the poultry industry has great potential, its growth is often hindered by diseases influenced by factors like location, climate, and farming practices (5). These diseases lead to significant production losses and require extensive vaccination programs. Endemic diseases and new viral strains continue to pose serious threats, making ongoing surveillance crucial for effective prevention (6–8). A 2011 World Bank study showed that a few diseases cause most livestock losses globally. The top three diseases account for about 80% of total losses for each species group, highlighting the potential for significant progress by targeting these priority diseases. For poultry, the top diseases include HPAI, Infectious bronchitis virus (IB), LPAI, and NDV, among others, causing substantial livestock losses (9).

In Kazakhstan’s poultry-breeding sector, veterinary specialists design vaccination programs tailored to the production volume and prevailing epizootic situation. These programs can be categorized into two main types: systematic and emergency. Systematic vaccination programs represent ongoing routine vaccination protocols, while emergency vaccination programs are implemented as a rapid response to disease outbreaks or situations with a heightened risk of disease introduction or emergence (10).

The dynamic poultry industry encounters intensified obstacles where productivity is crucial for sustainability. In this evolving landscape, producers are compelled to maximize output in a setting characterized by elevated disease prevalence, dense farm populations, restricted laboratory analysis, and antibiotic constraints (11). These challenges underscore the need for comprehensive disease management strategies that integrate vaccination programs, robust biosecurity practices, and informed poultry management techniques.

Between 2019 and 2024, the global poultry industry faced significant challenges due to various disease outbreaks. According to the WOAH, HPAI was the most prevalent disease, with 489 reported cases worldwide. Germany, Hungary, India, Poland, Russia, Bulgaria, and Moldova were among the most affected countries. Newcastle disease also posed a threat, with 52 cases reported globally, primarily in Russia and Sweden. Other notable diseases included Newcastle disease (ND) with 52 cases reported, primarily in Russia and Sweden, and avian infectious bronchitis in Hong Kong. Additionally, fowl typhoid, turkey rhinotracheitis, avian chlamydiosis, and other diseases posed regional threats (12).

The industrial poultry sector in Kazakhstan began to develop in 1965 (13). Today, poultry farming in Kazakhstan is growing, with expanding the bird population and production capacity. While this growth benefits the economy and food security, challenges remain. The industry relies heavily on imports, making it vulnerable to currency fluctuations. Additionally, limited access to subsidies and knowledge hinders small and new farmers (14). As of December 2023, the poultry stock in Kazakhstan amounted to 56.5 million heads (15). Key pathogens of infectious diseases in the sector include NDV, HPAI, and occasional cases of Avian Metapneumovirus (aMPV), IBDV, and IBV (12, 16–21).

We used a comprehensive approach to examine common poultry diseases in Kazakhstan. To understand the disease landscape, we analyzed official WOAH data and reviewed the scientific literature. Polymerase chain reaction (PCR) and serology analysis data from poultry farms were also collected. Moreover, a survey of veterinary specialists provided insights into on-farm realities, including vaccination strategies and biosecurity practices.

Through this combined approach, we aim to present a detailed overview of the current epidemiological situation in the poultry sector and to identify the factors influencing disease dynamics. By integrating these findings with a review of existing control measures and farming practices, this study seeks to facilitate informed decision-making regarding poultry health management in Kazakhstan.

## 2 Materials and methods

### 2.1 Study area

**Figure 1:**
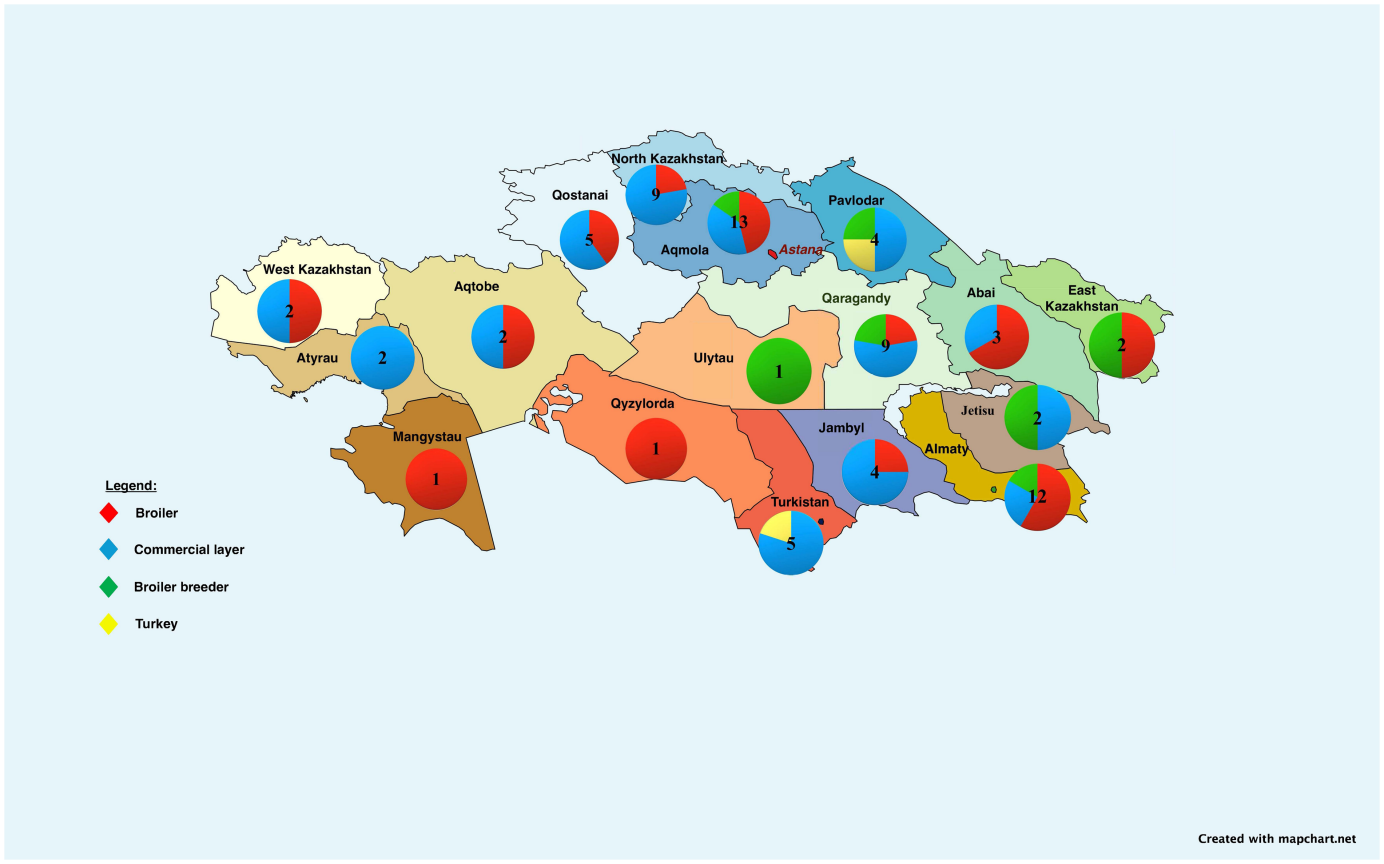
Regional map of Kazakhstan showing distribution and quantity of poultry farms by production focus: broiler, commercial layer, broiler breeder, and turkey farms. Some companies may operate multiple types of farms; quantities displayed represent total farm locations, regardless of farm type ownership. Further details are in the Supplementary material. The map was created with mapchart.net.

Kazakhstan is the largest and leading republic in Central Asia, covering 2.7 million square kilometers (22–24). In 2022, the country produced 5,028.5 million chicken eggs, exceeding domestic demand. By November 2023, production was 4,919.9 million eggs, or 98% of the need. Kazakhstan operates 37 egg farms, 27 meat farms, 8 broiler breeder farms, and 2 turkey farms (Figure 1). Additionally, poultry meat production reached 328.6 thousand tons in 2023, but domestic demand was not fully met (74%). The government plans 29 projects to reduce import reliance by 2027. In the first 11 months of 2023, Kazakhstan exported 186.6 million eggs, with significant exports to Afghanistan (114 M eggs), Kyrgyzstan (70 M eggs), and smaller quantities to Russia (2.6 M eggs). Additionally, Kazakhstan exported 28.2 thousand tons of poultry meat to Russia (19 thousand tons), Kyrgyzstan (7.5 thousand tons), Belarus (653 tons), and Uzbekistan (611.6 tons) (25, 26).

### 2.2 Study design and data collection

**Figure 2:**
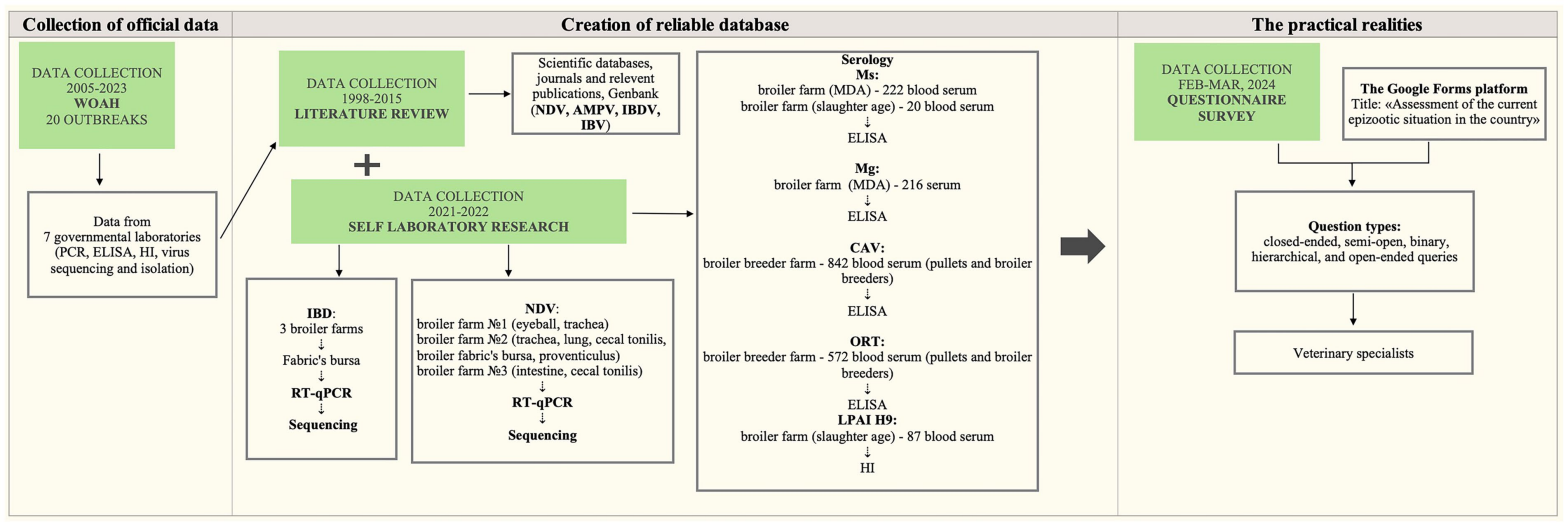
Summary of the methodology used and critical points.

A cross-sectional study was designed to estimate the prevalence of infectious diseases in the country. The study followed these steps to capture the situation accurately: (i) Collection of official data that government laboratories have provided to WOAH; (ii) Creation of a reliable database combining information collected through existing data and own laboratory research; (iii) Revealing the practical realities through conducting of a questionnaire for veterinary specialists at poultry farms. All stages of work and the corresponding key points are summarized in Figure 2.

#### 2.2.1 Sample collection

The Flinders Technology Associates filter paper (FTA^®^ Cards) is used to collect Real-Time PCR (RT-PCR) samples. Samples are stored and transported at room temperature in a dry place, accompanied by a sample submission sheet with all relevant information (27).

Each flock’s management and vaccination records were reviewed to correlate serological results with flock history. Sample selection criteria were based on random sampling to ensure unbiased representation of the target population.

Serum samples were collected from various broiler farms and broiler breeder farms selected based on geographic diversity, reported health issues and vaccination status. Day-old chicks were sampled to measure maternal-derived antibodies (MDA) for MG and MS. To investigate the cause of high mortality at slaughter age, samples were examined for MS and LPAI H9N2 on non-vaccinated broilers. To determine the immunological status and baseline antibody titers for CAV and ORT, serum samples were collected throughout the production period. For CAV, samples were taken from 3 to 406 days, with chickens vaccinated at 42 days of age using Nobilis CAV P4 (MSD Animal Health). For ORT, samples were collected from 40 to 406 days from non-vaccinated chickens. The data included four results from three different poultry houses.

#### 2.2.2 RNA extraction and virus detection

RT-PCR reaction and sequencing were performed in Expert Laboratory Ltd. in Moscow, Russia, and HN Veterinary Biotechnology Laboratory Research and Consultancy Ltd. in Turkey. Commercial kits, specifically KYLT^®^ IBDV GENOGROUP A3 (VERY VIRULENT) and Kylt^®^ Paramyxovirus 1 Pathotyping Real-Time RT-PCR Detection kit were used according to the manufacturer’s instructions.

#### 2.2.3 Sequencing and phylogenetic analysis

Sequencing was conducted by the Sanger sequencing method (SSM) (28), targeting the F protein gene for Newcastle Disease Virus (NDV) and the VP2 gene for Infectious Bursal Disease Virus (IBDV). For phylogenetic analysis, sequences from this study, including our newly obtained sequences and those received from GenBank, were combined into a FASTA file format. Sequences were initially aligned using Clustal Omega (29) to ensure accurate sample comparison. The aligned sequences were then analyzed using MEGA v11.0.10, where phylogenetic trees were constructed utilizing the Maximum Likelihood (ML) method (30).

#### 2.2.4 Antibody detection and serological analysis

Serum antibody detection was performed using commercially available indirect enzyme-linked immunosorbent assays (ELISAs) and a hemagglutination inhibition (HI) test. Specifically, Biochek ELISA kits (CK115 MS, CK114 MG, CK126 CAV, CK108 ORT) [BioChek, Reeuwijk, Netherlands] and IDEXX MS Ab Test 99-06728 [IDEXX (IDEXX Laboratories, Inc., Westbrook, ME, United States)] were employed following the manufacturers’ instructions. A HI reaction was performed using Royal GD VLDIA113 AI inactivated H9N2 antigen.

The analysis aimed to identify patterns in antibody responses and possible associations with farm management practices and vaccination protocols.

Descriptive statistics of serological results from ELISA and HI tests were performed using XLSTAT software to characterize the distribution of antibody titers. These statistics included minimum, maximum, range, quartiles (1st, median, and 3rd), mean, and standard deviation.

Inferential statistics (two-sample *t*-test and *z*-test, ANOVA) were employed to compare antibody titers between different groups.

#### 2.2.5 Questionnaire survey

A questionnaire was developed for veterinary specialists at poultry farms to gather insights into the current conditions within the Republic of Kazakhstan. A 25-question survey, titled “Assessment of the current epizootic situation in the country,” was hosted on the Google Forms platform. Information which we collected from this survey: (i) type of production; (ii) location of farm; (iii) permanent number of livestock (approximate number of heads present at the same time at all production sites); (iv) occurrence of the following avian infectious diseases during the last year (from 01.01.2023 to 31.12.2023): NDV, IBV, LPAI H9, HPAI H5, IBD, MG, MS, Fowl Adenoviruses (FAdV), Reovirus (REO), Infectious laryngotracheitis (ILT), CAV, Egg drop syndrome (EDS), aMPV, Avian encephalomyelitis (AE), сoccidiosis, other bacterial infectious (Salmonella spp., *Escherichia coli*, Pseudomonas spp., Clostridium spp., etc.), other diseases; (v) evaluation of the prevalence of diseases on a scale from 1 to 10 (where 1 is never occurred and 10 is extremely common); (vi) presence of monitoring of infectious diseases; (vii) preferences in infectious disease monitoring methods; (viii) presence of regular vaccination programs; (ix) diseases which prevented by vaccination; (x) preferences in vaccines suppliers. In addition to the survey responses, veterinary specialists were requested to provide vaccination protocols, biosecurity measures, and information about farm management practices to gain a comprehensive understanding of disease control strategies across farms. The survey included responses from farms located in 7 out of 17 regions of Kazakhstan. The link for the questionnaire is https://forms.gle/6YtEpE5pfdBVTksd8.

## 3 Results

### 3.1 WOAH data

The World Organization for Animal Health (WOAH) provided data about registered avian diseases in Kazakhstan from 2005 to 2023. A total of 27 outbreaks were reported, 20 in domestic birds and 7 in wild animals. Among domestic birds, only Avian Influenza (H5N1, H5N2, H5N8, and H5N untyped) and Newcastle Disease (*Avian orthoavulavirus* 1) were detected. The analysis of quantitative data was conducted through descriptive statistics (Table 1) (10).

**Table 1:** Descriptive statistics for outbreaks of avian influenza (quantitative data).

Disease	Statistic	Susceptible	Positive samples	Deaths	Killed and disposed	Prevalence
AI (H5(N untyped), H5N1, H5N2, H5N8)	Minimum	48,0	2,0	2,0	0,0	0,22%
	Maximum	602,784,0	67,957,0	67,957,0	534,827,0	100%
	1st quartile	260,0	76,0	76,0	52,0	2,4%
	Median	2,556,0	235,0	235,0	260,0	14,3%
	3rd quartile	6,562,0	376,0	376,0	2,128,0	40,5%
	Sum	678,877,0	72,438,0	72,438,0	550,138,0	-
	Mean	39,933,9	4,261,1	4,261,1	32,361,1	31,9%
	Standard deviation (n)	140,867,6	15,927,1	15,927,1	125,625,4	33,2%
NDV (APMV-1)	Minimum	31,0	1,0	0,0	48,0	0,4%
	Maximum	467,0	31,0	31,0	467,0	100%
	1st quartile	145,5	3,0	0,0	154,0	0,7%
	Median	260,0	5,0	0,0	260,0	1,1%
	3rd quartile	363,5	18,0	15,5	363,5	50,5%
	Sum	758,0	37,0	31,0	775,0	-
	Mean	252,7	12,3	10,3	258,3	33,8%
	Standard deviation (n)	178,1	13,3	14,6	171,1	46,8%

Further details are available in the Supplementary material.

#### 3.1.1 Avian influenza (HPAI H5)

The median prevalence rate was 14.3% (95% CI: 16.7%–48.16%), with instances ranging from 0.22 to 100%. Severe outbreaks, characterized by 100% prevalence, contributed significantly to the observed variability (standard deviation = 33.2%). Mortality (deaths) and eradication efforts (killed and disposed) were considerable, with totals of 72,438 deaths and 550,138 culled birds, respectively.

Association coefficients calculated during the study for HPAI:

- Pearson’s Phi: 0.506 (Moderate Association);
- Goodman and Kruskal Gamma: 0.502 (Moderate Association);
- Kendall’s Tau: 0.141 (Weak Association);
- Stuart’s Tau: 0.051 (Very Weak Association);
- Somers’ D (R/C): 0.069 (Very Weak Association);
- Somers’ D (C/R): 0.287 (Weak Association).

### 3.2 Past outbreaks of poultry infections in Kazakhstan (1988–2015)

Several outbreaks of avian influenza have been documented in Kazakhstan over the years. Avian influenza virus A/H11N9 was isolated from farmed turkeys near Almaty in 2004. These isolates were classified as low-pathogenic avian influenza viruses (31). Subsequent years saw additional outbreaks. HPAI H5N1 virus strain A/domestic goose/Pavlodar/1/2005 (H5N1) was isolated in Pavlodar for the first time in Kazakhstan in 2005 (32). Subsequently this virus was found in rural poultry near Astana (GenBank: FJ390030.1, FJ390032.1, FJ390029.1, FJ390028.1, FJ390031.2). The same year, two strains of HPAI H5N1 were isolated during epizootic outbreak among poultry in North Kazakhstan and Qaragandy regions (GenBank: MN700651.1, MN880476.1). More recently, in 2020, outbreaks of the HPAI H5N8 virus (A/Aviafauna/Kazakhstan/HA/2020) have occurred, with nine isolates identified in North Kazakhstan and eight isolates found in Almaty (33).

The analysis conducted in Kazakhstan from 1998 to 2005 investigated NDV outbreaks affecting chicken flocks in several regions, including Almaty, Taldykorgan, and Astana. Researchers isolated 28 NDV strains, all highly virulent based on international criteria. These strains had specific characteristics in the fusion protein indicative of virulence and a high Intracerebral Pathogenicity Index (ICPI) (0.7 or greater). Notably, 24 isolates were classified as velogenic, characterized by a short mean death time (less than 60 h) and a very high ICPI (average 1.5). The remaining 4 strains were mesogenic, with a similar death time but a lower ICPI (less than 1.5) (16).

According to Orynbayev et al. (17), multiple outbreaks of Newcastle disease were documented in various regions of Kazakhstan. Significant losses were reported, including: in 2010, over 2,000 birds died in a poultry farm in the Ili district, Almaty region; in October 2012, more than 900 birds died in rural flocks of Aksuat, North Kazakhstan region; and in June 2013, mass mortality affected poultry in Otar and Matybulak, Jambyl region. The outbreaks in Almaty, Jambyl, and North Kazakhstan regions in 2010, 2012, and 2013 were primarily linked to genotype VIIb. However, a separate study identified genotype VIId in a vaccinated poultry farm, highlighting the complex epidemiological dynamics of the disease (17).

A study conducted in Kazakhstan between 2011 and 2012 investigated the presence of aMPV in poultry. The study employed an ELISA to test 317 serum samples collected from various poultry farms across the country. Notably, the ELISA revealed high antibody titers (average 22,859 ± 4,133) in unvaccinated chickens, suggesting widespread aMPV exposure. These findings led researchers to suspect aMPV infection in the tested birds (18).

The IBDV in Kazakhstan was represented by the strain IBDVKaz03-2014, which was characterized by the VP2 and VP1 genes (19).

In 2007, a single isolation of the Arkansas genotype of IBV was found in Kazakhstan. Information confirming whether the Arkansas live vaccine has ever been administered to these flocks is not available. Partial sequence data for the S1 glycoprotein gene of infectious bronchitis virus (Isolate KZ/14/2007) were obtained from the GenBank database (HQ840499.1) (20, 21).

### 3.3 Current epidemics of avian viral diseases in the Republic of Kazakhstan

#### 3.3.1 Infectious bursal disease virus

**Figure 3:**
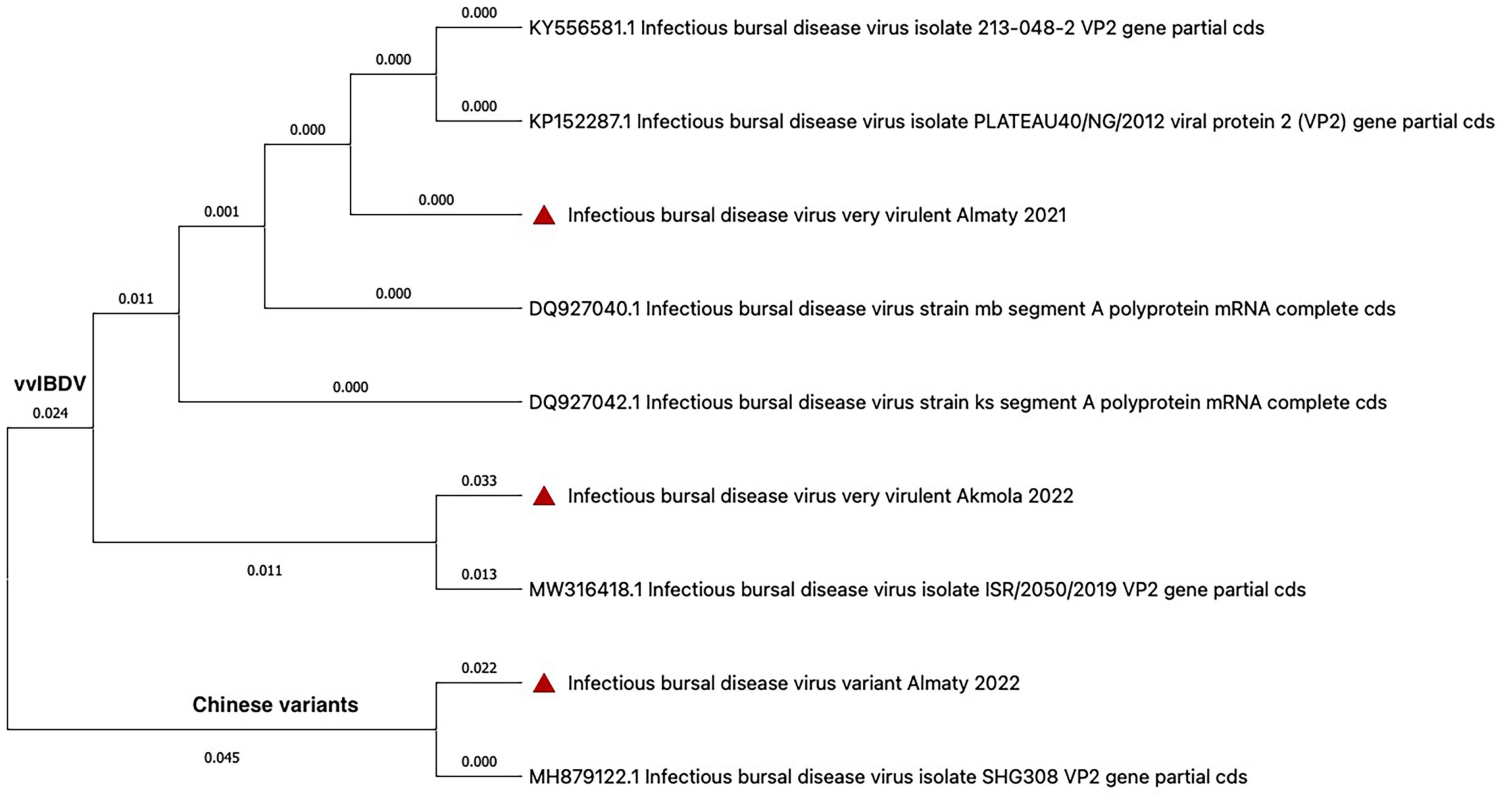
The phylogenetic tree of VP2 gene sequences shows isolates from Kazakhstan marked with red triangles.

In June 2021, the outbreak of IBD was confirmed from a poultry farm in the Almaty region. Sequencing of the VP2 gene fragment revealed 100% sequence identity with known isolates MB DQ927040, BG, PLATEAU40/NG/2012 KP152287, and 213–048-2 KY556581.1 (34, 35). In July 2022, another IBD outbreak occurred in the same region. Sequence analysis of the VP2 gene fragment confirmed a 100% match with the SHG308 isolate, a known immunosuppressive variant from China (MH879122.1, China, 2019) (36). Additionally, in June 2022, a very virulent Gumboro disease virus was detected in a poultry farm in the Akmola region. Genetic analysis revealed a close relationship to other very virulent strains (95.7% or greater) (37, 38). A phylogenetic tree was constructed using the VP2 gene sequences (Figure 3) to visualize the relationships between the IBDV strains identified in this study and known isolates.

#### 3.3.2 Newcastle disease virus

**Figure 4:**
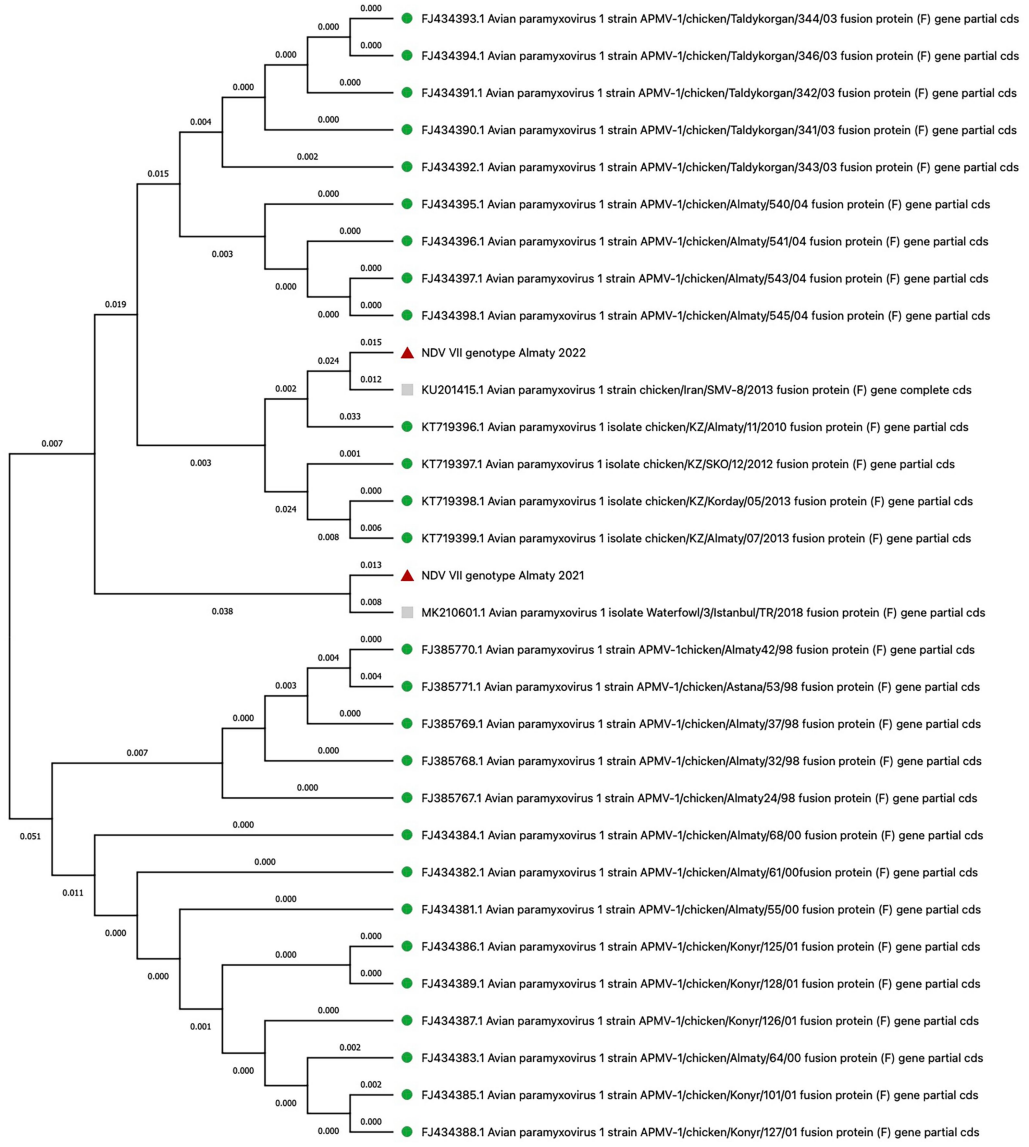
Phylogenetic analysis of NDV isolates based on fusion protein (F) gene sequences. Red triangles denote isolates from this study, grey squares indicate isolates with genetic similarity to those in our study, and green circles represent isolates from Kazakhstan (1998–2013).

In several poultry farms, samples collected during severe outbreaks revealed the presence of NDV genotype VII. In August 2021, three samples from the Almaty region were genetically identical and showed a 98% nucleotide identity with genotype VII subtype VIIi isolates, such as Waterfowl/1/Istanbul/TR/2018 (MK210599.1), Backyard_chicken/1/Istanbul/TR/2018 (MK210601.1), and PHL159057 (MH371070.1) (39). In April 2022, NDV was again identified in the same region, and sequence analysis confirmed genotype VII.2. Although the specific gene sequences were not provided, phylogenetic trees constructed by the HN Veterinary Biotechnology Laboratory Research and Consultancy Ltd. in Turkey supported this genotype assignment. Furthermore, in June 2022, NDV was detected in a poultry farm in the Akmola region. Three samples were genetically identical and had a 97.4% similarity to previously reported domestic and genotype VII isolates, such as chicken/Iran/SMV-8/2013 (KU201415.1) and others (40, 41). A phylogenetic tree was constructed based on the fusion protein (F) gene to visualize the evolutionary relationships between the NDV strains identified in this study and previously known isolates from Kazakhstan, spanning from 1998 to 2013 (Figure 4; Supplementary material) (16, 17).

### 3.4 Serological survey results

#### 3.4.1 Mycoplasma synoviae

Antibodies for MS were detected in samples of day-old chicks as well as in chicks at slaughter age collected from various farms in the Almaty and Jambyl regions between March to June 2022. The results are presented in Supplementary materials and summarized in Table 2 and detailed statistics are provided in Table 3.

**Table 2:** ELISA results for MS in serum samples from broiler flocks.

Egg supplier	Location of farm	Age, days	Sample size	Mean titer	Positive	Negative	CV, %	Min titer	Max titer
Uzbekistan	Almaty	1	18	1,252	9	9	119	5	4,742
Uzbekistan	Almaty	1	18	909	8	10	105	2	3,784
Uzbekistan	Almaty	1	18	2,461	15	3	100	75	8,163
Uzbekistan	Almaty	1	18	1,674	14	4	82	404	5,149
Uzbekistan	Almaty	1	18	1,162	11	7	92	167	4,079
Turkey	Almaty	1	18	21	0	18	243	2	228
Turkey	Almaty	1	18	9	0	18	67	1	20
Uzbekistan	Almaty	1	18	1,286	12	6	102	101	4,472
Uzbekistan	Almaty	1	18	1,137	12	6	81	201	3,436
Uzbekistan	Almaty	1	18	1,463	11	7	164	188	10,697
Uzbekistan	Jambyl	40	20	1775	14	6	47,1	615	3,583
Uzbekistan	Almaty	1	21	623	6	15	132	1	3,679
Uzbekistan	Almaty	1	21	457	6	15	85	52	1,656

**Table 3:** Descriptive statistics of ELISA results for MS.

Egg supplier	Statistic	Mean titer	CV, %	min titer	max titer	Positivity, %
Uzbekistan	Minimum	457	47	1	1,656	29
	Maximum	2,461	164	615	10,697	83
	Range	2004	117	614	9,041	54
	1st quartile	1,023	84	29	3,631	47
	Median	1,252	100	101	4,079	61
	3rd quartile	1,569	112	195	4,946	69
	Mean	1,291	101	165	4,858	58
	Standard deviation (n)	532	29	182	2,380	17
Turkey	Minimum	9	67	1	20	0
	Maximum	21	243	2	228	0
	Range	12	176	1	208	0
	1st quartile	12	111	1	72	0
	Median	15	155	2	124	0
	3rd quartile	18	199	2	176	0
	Mean	15	155	2	124	0
	Standard deviation (n)	6	88	1	104	0

#### 3.4.2 Mycoplasma gallisepticum

In April 2022, blood serum samples were collected at a poultry farm in the Almaty region to monitor maternal antibodies against MG in day-old chicks. The results are presented in Table 4 and in Supplementary materials, with comprehensive descriptive statistics available in Table 5.

**Table 4:** ELISA results for MG in serum samples from broiler flocks.

Egg supplier	Location of farm	Age, days	Sample size	Mean titer	Positive	Negative	CV, %	Min titer	Max titer
Uzbekistan	Almaty	1	18	93	1	17	292	1	1,175
Uzbekistan	Almaty	1	18	761	5	13	175	9	4,029
Uzbekistan	Almaty	1	18	918	8	10	123	162	5,076
Uzbekistan	Almaty	1	18	541	4	14	142	28	3,343
Uzbekistan	Almaty	1	18	830	7	11	137	68	4,852
Turkey	Almaty	1	18	8	0	18	75	1	20
Turkey	Almaty	1	18	9	0	18	89	9	32
Uzbekistan	Almaty	1	18	880	7	11	133	9	4,248
Uzbekistan	Almaty	1	18	974	8	10	129	1	3,943
Uzbekistan	Almaty	1	18	2,106	9	9	141	11	8,816
Uzbekistan	Almaty	1	18	458	6	13	106	16	1,398
Uzbekistan	Almaty	1	17	594	4	13	168	1	3,829

**Table 5:** Descriptive statistics of ELISA results for MG.

Egg supplier	Statistic	Mean titer	CV, %	Min titer	Max titer	Positivity, %
Uzbekistan	Minimum	93	106	1	1,175	6
	Maximum	2,106	292	162	8,816	50
	Range	2013	186	161	7,641	44
	1st Quartile	554	130	3	3,465	25
	Median	796	139	10	3,986	36
	3rd Quartile	909	162	25	4,701	43
	Mean	816	155	31	4,071	33
	Standard deviation (n)	498	50	48	2008	12
Turkey	Minimum	18	75	1	20	0
	Maximum	18	89	9	32	0
	Range	0	14	8	12	0
	1st Quartile	18	79	3	23	0
	Median	18	82	5	26	0
	3rd Quartile	18	86	7	29	0
	Mean	18	82	5	26	0
	Standard deviation (n)	0	7	4	6	0

#### 3.4.3 Chicken anemia virus

**Figure 5:**
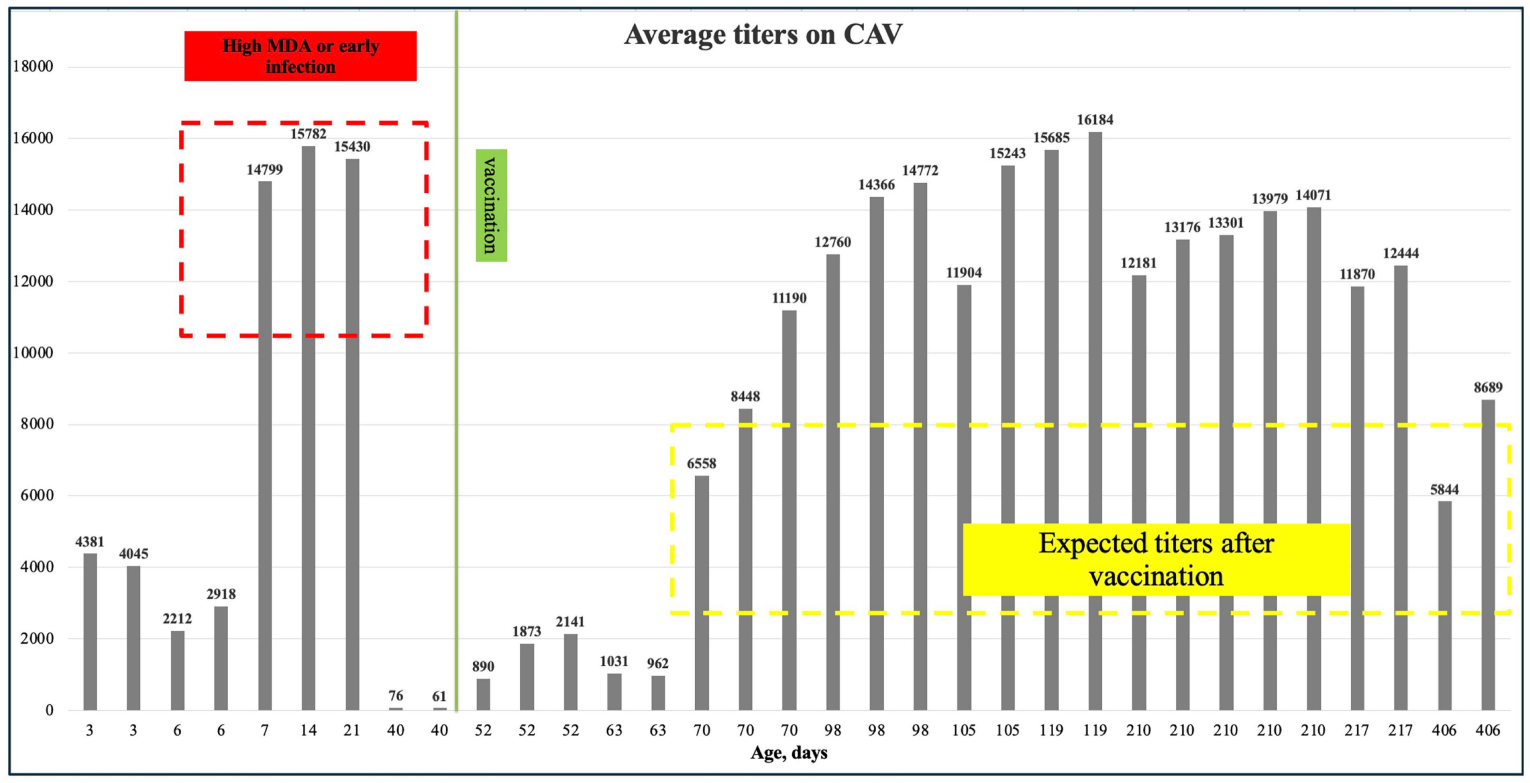
Mean CAV titers, where the red dashed line indicates high maternal antibodies, the green line marks the period when CAV P4 vaccination was administered, and the yellow dashed line shows the expected titers after vaccination.

This study examined the levels of antibodies against CAV in a breeder farm in Almaty region between May and July 2022. According to the Biochek interpretation and application of the results manual, expected antibody levels after vaccination (4–6 weeks) should be between 3,000 and 8,000. As shown in Table 6 and Figure 5, the average antibody levels before and after vaccination ranged from 6,558 to 16,184, significantly exceeding the expected range of 3,000 to 8,000. The *p*-values for both Q1-Inadequate vaccination (titer under 3,000) (*p* = 0.045) and Q1- Suspect titer infection (*p* < 0.0001) suggest a statistically significant relationship between these variables and antibody levels (Table 7).

**Table 6:** Descriptive statistics of ELISA results for CAV.

Statistic	CV, %	Mean titer	Min titer	Max titer	Positivity, %
Minimum	3	61	1	308	0
Maximum	205	16,184	15,228	18,971	100
Range	202	16,123	15,227	18,663	100
1st Quartile	23	2,918	363	7,068	94
Median	33	11,870	3,390	16,589	100
3rd Quartile	54	14,071	6,109	17,195	100
Mean	45	9,069	4,182	12,337	**88**
Standard deviation (n)	40	5,664	4,114	6,040	27

The high variability in titers and the majority positivity rate (88%) suggest that vaccination was effective in the majority of cases. The value 88% is highlighted in bold to indicate the high variability in titers and the majority positivity rate, demonstrating the effectiveness of vaccination in the majority of cases.

**Table 7:** Regression analysis of antibody levels for CAV.

Source	Value	Standard error	t	Pr > |t|	Lower bound (95%)	Upper bound (95%)	*p*-values signification codes
Intercept	5,881,500	375,593	15,659	**<0,0001**	5,143,167	6,619,833	***
Q1-inadequate vaccination	−1,178,757	585,319	−2,014	**0,045**	−2,329,367	−28,148	*
Q1-suspect titer infection	8,359,711	403,287	20,729	**<0,0001**	7,566,937	9,152,485	***

Bold values indicate p-values that are statistically significant, highlighting a meaningful relationship between the variables and antibody levels. Significance codes: ***(*p* < 0.001), **(*p* < 0.01), *(*p* < 0.05), (*p* < 0.1), ° (*p* < 1).

#### 3.4.4 Ornithobacterium rhinotracheale

**Figure 6:**
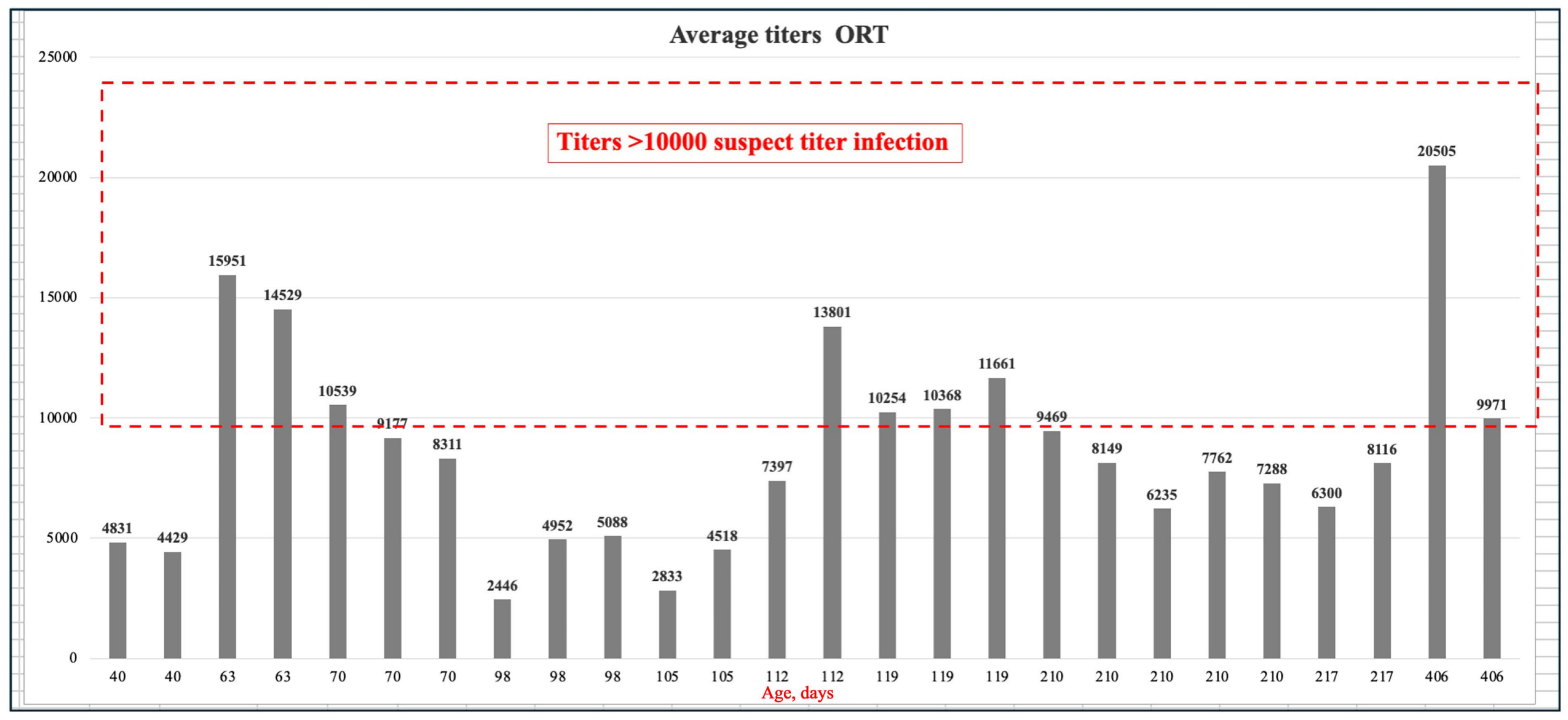
Mean ORT titers, where the red dashed line indicates the titers >10,000.

This study investigated the presence of antibodies against ORT in a non-vaccinated breeder farm in Almaty region between May and July. According to the Biochek manual, no antibodies should be detectable in non-vaccinated birds, and titers exceeding 10,000 are considered indicative of a suspect infection. The results revealed significantly elevated antibody levels, ranging from 2,446 to 20,505 (Figure 6). The mean of suspect titers (15098.763) is considerably higher than the mean of acceptable titers (5026.139). A *p*-value <0.0001 indicates that the difference in mean titers is statistically significant, meaning it is unlikely to have occurred by chance. Detailed results and all collected data are available in the Supplementary materials. A summary of the statistical analysis of these results is presented in Table 8.

**Table 8:** Summary statistic analysis of ELISA results for ORT.

Variable	Observations	min titer	max titer	Mean titer	Standard deviation (n)
Suspect titer infection	198	10,018	32,943	15,099	4,246
Acceptable titers	374	84	9,995	5,026	2,679

A *p*-value < 0.0001 indicates that the difference in mean titers is statistically significant, meaning it is unlikely to have occurred by chance.

#### 3.4.5 Low pathogenic avian influenza

In July 2022, a poultry farm in the Akmola region provided data regarding the seroprevalence of LPAI H9. Out of 86 samples analyzed, 7 were negative, and 80 were positive for LPAI H9 antibodies. The antibody levels in birds at slaughter age ranged from 2.7 to 4.5 Log2 (Table 9).

**Table 9:** HI results for LPAI H9 with standard deviation (SD).

Type	Number of flocks	Sample size	H9 S/N ratio (mean Log2 ± SD)	CV (%) (mean)	Positivity
broilers	4	86	3,75 ± 0.712	42	91.9%

### 3.5 Questionnaire survey (practical realities)

**Figure 7:**
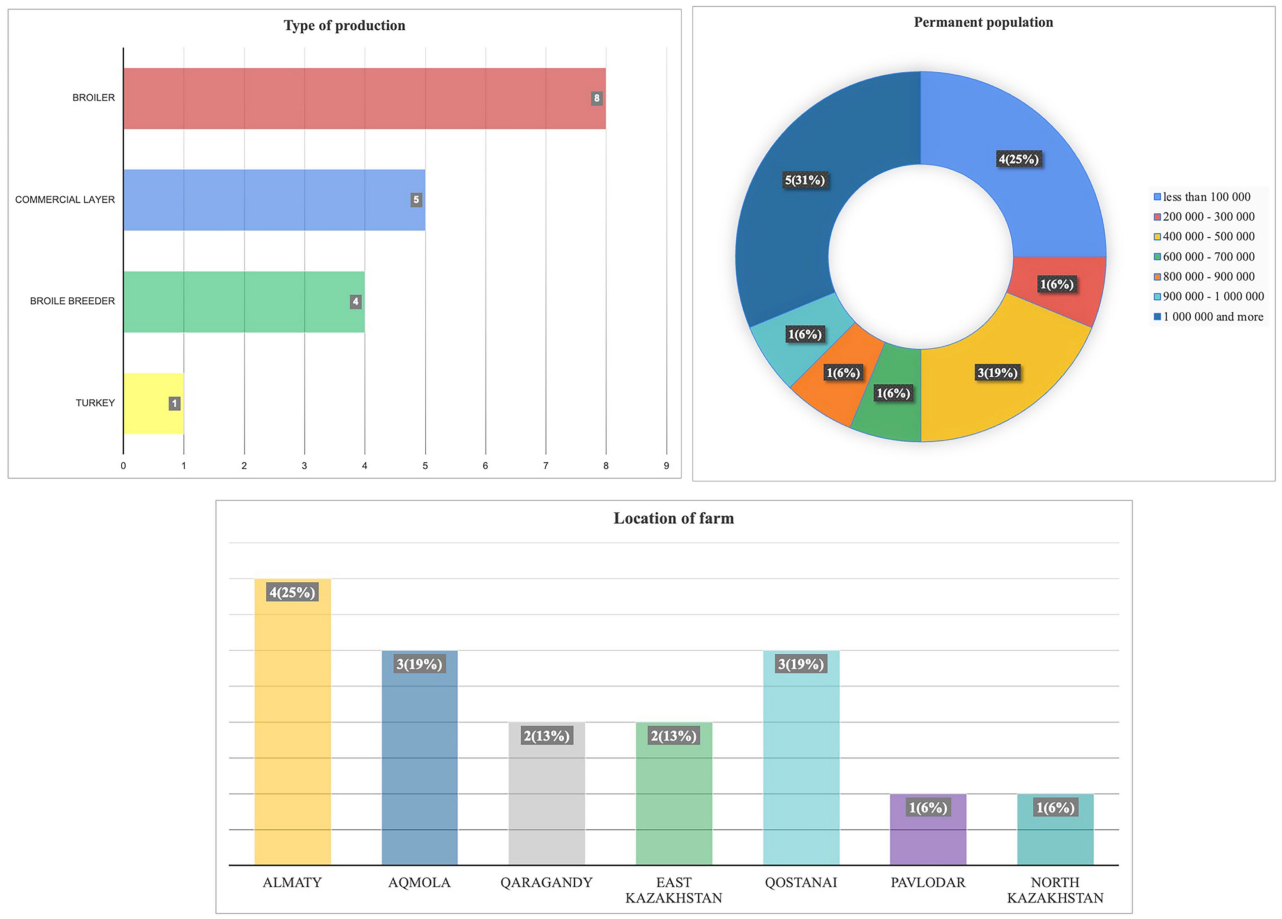
**(A)** Types of production, **(B)** populations, and **(C)** locations of farms that participated in the questionnaire survey.

Between February 29 and March 13, 2024, a survey titled “Assessment of the current epizootic situation in the country” was conducted at 16 poultry farms. The farms raised various poultry types, including broilers (50%), commercial layers, broiler breeders, and turkeys. The survey revealed a geographically diverse distribution of participating farms. The Almaty region had the highest representation at 25%, followed by the Aqmola and Qostanai regions, each contributing 18% of respondents. The North Kazakhstan and Pavlodar regions had the lowest participation rates, each representing 6.3%. The questionnaire also highlighted the varied sizes of bird populations on the farms. Notably, 31% of farms housed over 1 million birds, while the next largest group (25%) consisted of farms with populations under 100,000 birds (Figure 7).

**Figure 8:**
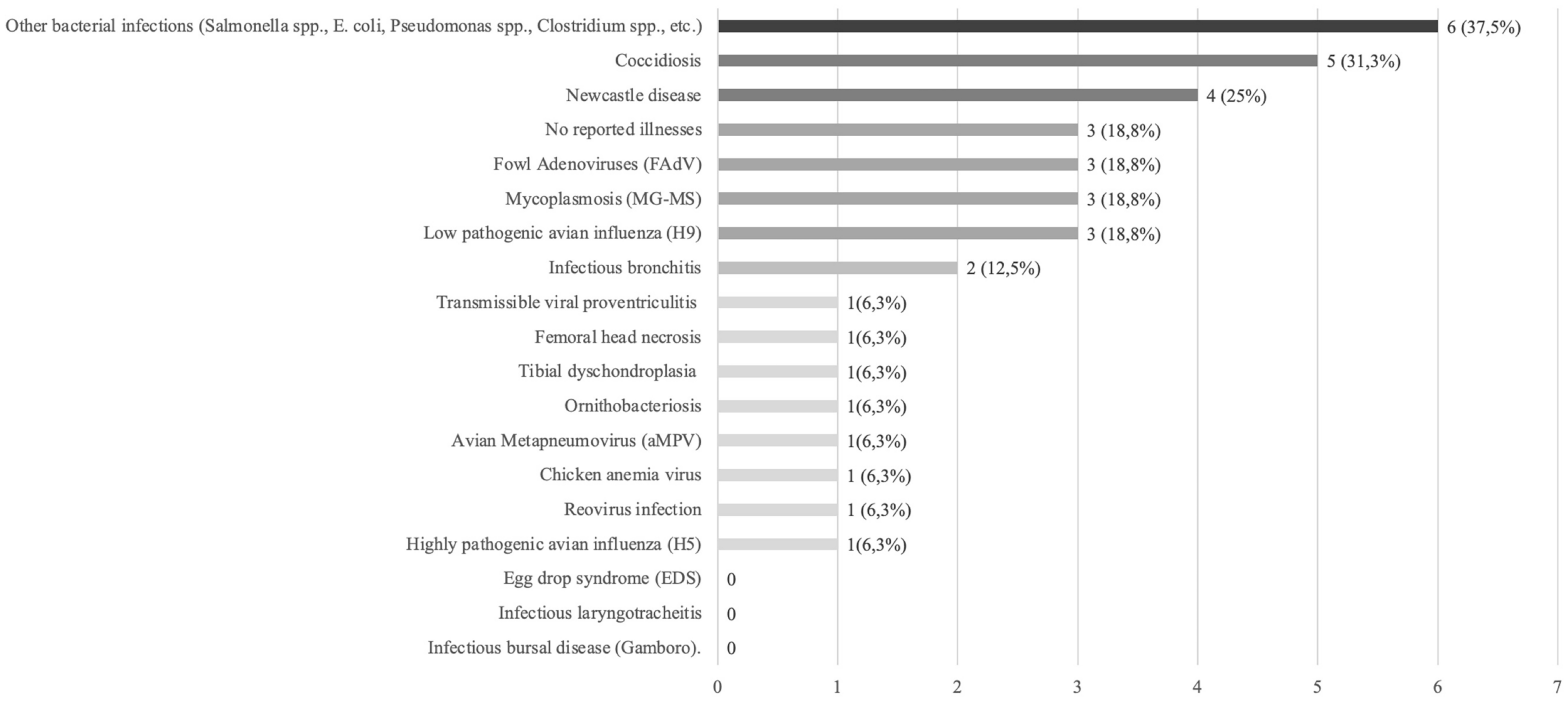
The distribution of various poultry infectious diseases.

The distribution of various poultry infectious diseases was monitored throughout 2023. Data analysis revealed that the most common diseases were other bacterial infections (Salmonella spp., *E. coli*, Pseudomonas spp., Clostridium spp., etc.) at 37.5%, coccidiosis at 31.3%, and Newcastle disease at 25% (Figure 8).

The prevalence of diseases was evaluated on a scale from 1 to 10, where level 1 indicates that the disease never occurred and level 10 signifies extremely common occurrence. Diseases that reached the highest prevalence level (level 10) were identified as significant issues in Kazakhstan’s poultry industry. For example, NDV was a primary concern on four farms: two in the Almaty region, one in Aqmola, and one in Qostanai. IBV was observed on two farms, both in the Almaty region. LPAIV was detected on three farms: two in the Almaty region and one in Qostanai. FAdV was significant on one farm in the Almaty region, while aMPV was noted on another farm also in the Almaty region. Additionally, other bacterial diseases (including Salmonella spp., *Escherichia coli*, Pseudomonas spp., Clostridium spp., etc.) were prevalent at level 10 on three farms: two in the Almaty region and one in Aqmola. Refer to Supplementary materials for details.

**Figure 9:**
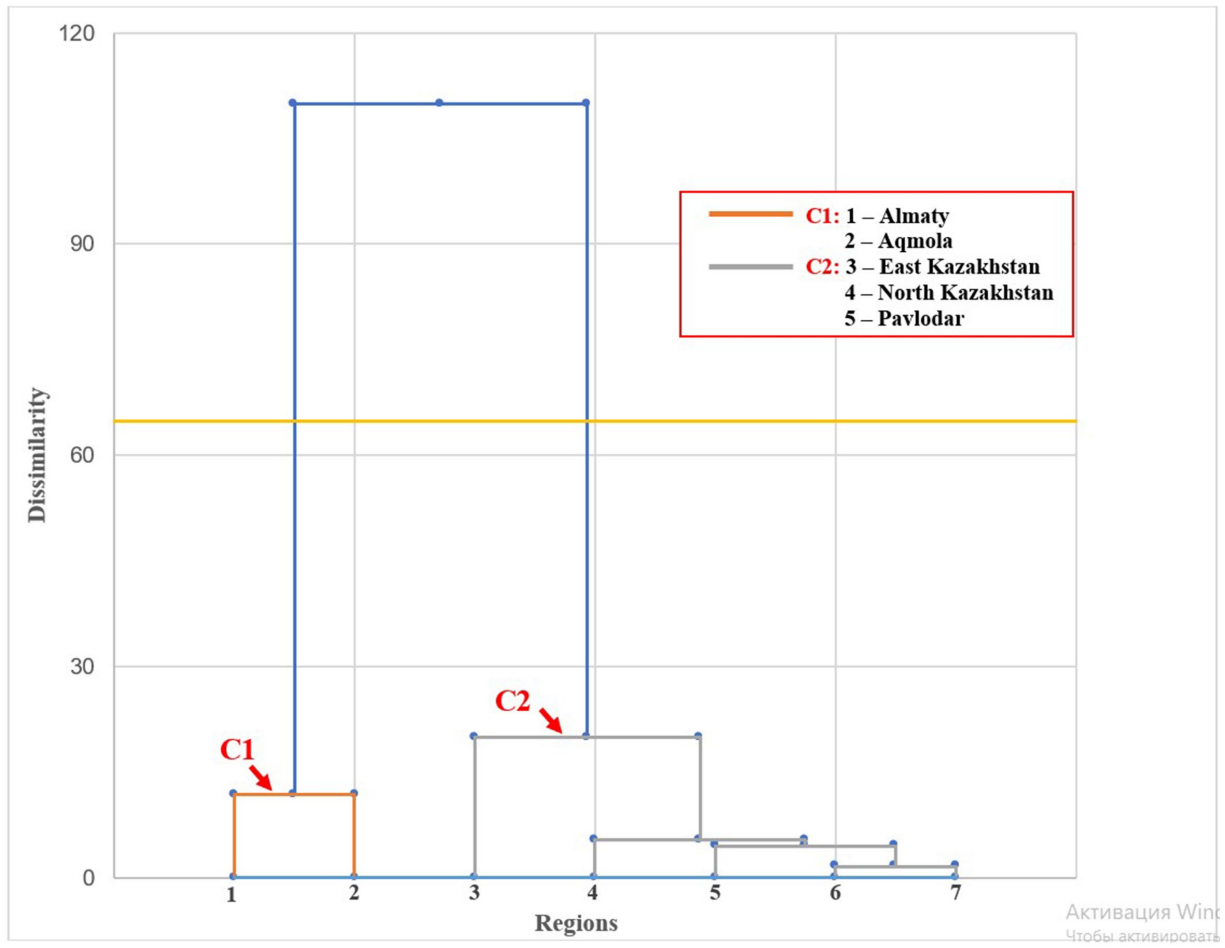
Hierarchical clustering of regions by disease prevalence.

To analyze the disease prevalence, average prevalence rates for each disease by region were calculated (Table 10), and a cluster analysis was performed to group the regions based on similarities in disease distribution. Based on the obtained data, two clusters were identified (Figure 9). Cluster 1 (C1): Almaty and Akmola exhibit a high degree of similarity in disease distribution, with a relatively low dissimilarity index of approximately 60. This suggests a shared pattern of disease spread across these two areas. Cluster 2 (C2): Kostanay shares some similarities with Almaty and Akmola, as evidenced by its inclusion in Cluster 2 at a higher level of dissimilarity. However, it also displays significant differences in disease spread compared to these regions. Additionally, the regions Karaganda, North Kazakhstan, East Kazakhstan, and Pavlodar form a separate cluster, which may indicate similar disease distribution trends among these regions.

**Table 10:** Average disease prevalence by region.

Cluster	Region	NDV	IBV	LPAI H9	HPAI H5	IBD	MG-MS	FadV	Reo	ILT	CAV	EDS	aMPV	Cocci-diosis	Other bac-terial inf.
C1	Almaty	5,5	6	5,5	4	3,8	3,25	5,8	2	1,5	1,8	1,8	3,3	3,8	6,5
	Aqmola	4,3	4,3	3,6	2,3	3,3	4	3	2,3	1,6	2,3	1,6	4,3	4,6	5
C2	East Kazakhstan	1	1	1	1	1,5	2,5	1	1,5	1	1	1	1	2,5	3,5
	North Kazakhstan	1	1	1	1	1	2	1	1	1	1	2	1	1	1
	Pavlodar	1	1	1	1	1	2	1	1	1	1	1	1	1	3
	Qaragandy	2	2,5	1	1	1	1	2	1,5	1	1,5	1	1	2,5	2,5
	Qostanai	4	3	4	1	1,3	1	1	1	1,7	1	1,7	1,7	2,3	4,3

**Figure 10:**
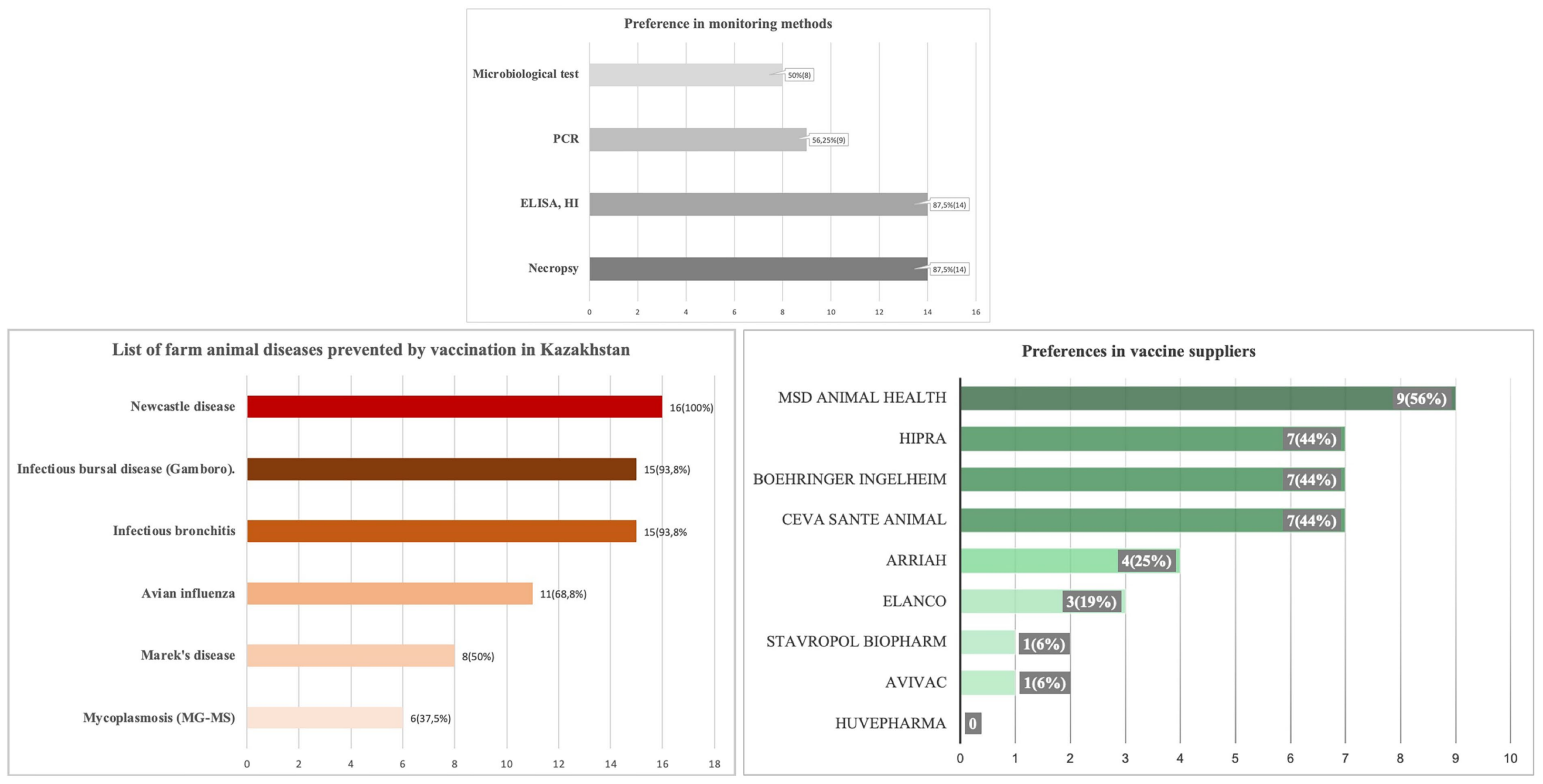
**(A)** Disease control measures, **(B)** vaccination practices, and **(C)** vaccine supplier preferences in poultry farms in Kazakhstan.

Infectious disease control is conducted across all poultry farms except for a single farm with a population off less than 100,000 birds. 14 out of 15 farms use necropsy, ELISA, and HI tests for diagnosis. Nearly half of these farms also use PCR and microbiological tests. All but one farm vaccinate their poultry systematically. NDV vaccination is prioritized by all farms, while IBD and IBV vaccinations are used by 15 farms. Conversely, only six farms undertake vaccination measures against mycoplasmosis. A survey was also conducted to assess supplier preferences for poultry vaccines in the Kazakhstan market. While there is a diverse range of suppliers, the survey identified MSD Animal Health as the leading choice. Ceva Santé Animale, Boehringer Ingelheim, and HIPRA tied for second place (Figure 10).

#### 3.5.1 Analysis of poultry vaccination schemes in Kazakhstan

In 2023, data were collected from seven broiler farms, two commercial layer farms, and one broiler breeder flock to evaluate vaccination practices.

##### 3.5.1.1 Broiler farms

Data analysis from broiler farms showed that most enterprises use similar vaccination protocols, including vaccination of day-old chicks in the incubator and subsequent vaccination in the field. However, significant differences were identified, dividing the farms into two main groups. The first group applies either inactivated vaccines against ND (e.g., the LaSota strain) administered in the incubator or live frozen vector vaccines (the D-26 strain). The second group exclusively uses live vaccines against ND, primarily based on lentogenic strains (Clone 30, LaSota, Avinew, B1, C2).

To protect against IB, most enterprises use the classical Massachusetts strain from line GI-1 (Ma5, H120, B-48, CR88) and its variants from line GI-13 (793B, 4/91). While many farms adhere to this practice, there are differences in strain selection and vaccination timing. Vaccination programs of broiler farms show significant similarities: almost all use live vaccines against Newcastle disease, employing lentogenic strains (Clone 30, LaSota), administered via spray, drinking water, or a combination thereof.

In addition to ND and IB, broiler farms administer vaccination once against LPAI and twice against IBD (Table 11).

**Table 11:** Summarized vaccination protocol on broiler farms in Kazakhstan.

Location	Disease	Strains	Type of vaccine	Delivery method	Note
Hatchery	ND	Clone 30, LaSota, Avinew, B1, C2	Live	Spray	ONE of these strains might be used
		+			
		Lasota	Inactivated	Subcutaneous	ONE of this type of vaccines or BOTH might be used. OR they are not used at all
		or			
		D-26	Live frozen vector vaccine	Subcutaneous	
	LPAIV	H9	Inactivated	Subcutaneous	Only in farms with HIGH PRESURE. Commonly in combination with Lasota
	IB	GI-1: (Ma5, H120, B-48, CR88)	Live	Spray	These strains might be used on the hatchery OR/AND on the field
		+			
		GI-13: (793B, 4/91)	Live	Spray	
	IBD	Faragher 52/70 (+vHVT013-69)	Live frozen vector vaccine	Subcutaneous	ONE of this type of vaccine might be used. OR they are not used at all, on this case IBD vaccines used only on the field **
		or			
		Winterfield 2,512	Immune-complex live IBD vaccine	Subcutaneous	
Field	ND	Clone 30, LaSota	Live	Spray+drinking water or just drinking water	Depending on field pressure farms vaccinate 1–2-3 times
	IBD	** 228E, Winterfield 2,512, M.B., GM 97	Live	Drinking water	ONE of these strains might be used. Vaccination frequency (1–2 times) depends on presence of hatchery vaccination
	IB	GI-1: (Ma5, H120, B-48, CR88)	Live	Spray	These strains might be used on the hatchery OR/AND on the field
		+			
		GI-13: (793B, 4/91)	Live	Spray	

### 3.6 Commercial layer and broiler breeder farms

The vaccination schemes across different poultry production types in Kazakhstan were examined, focusing on commercial layers (from Jetisu and Aqmola regions) and broiler breeders (Almaty region). A total of 13 diseases were included in the analysis. Statistical analysis of the vaccination schemes across these different poultry production types revealed no significant regional differences in vaccination frequencies. A chi-square test was conducted to assess the observed differences in vaccination frequencies for various diseases across the farms. The analysis revealed no statistically significant variation (χ^2^ = 33.20, df = 30, *p* = 0.3141), indicating that vaccination practices are similar across the egg-laying production systems.

**Figure 11:**
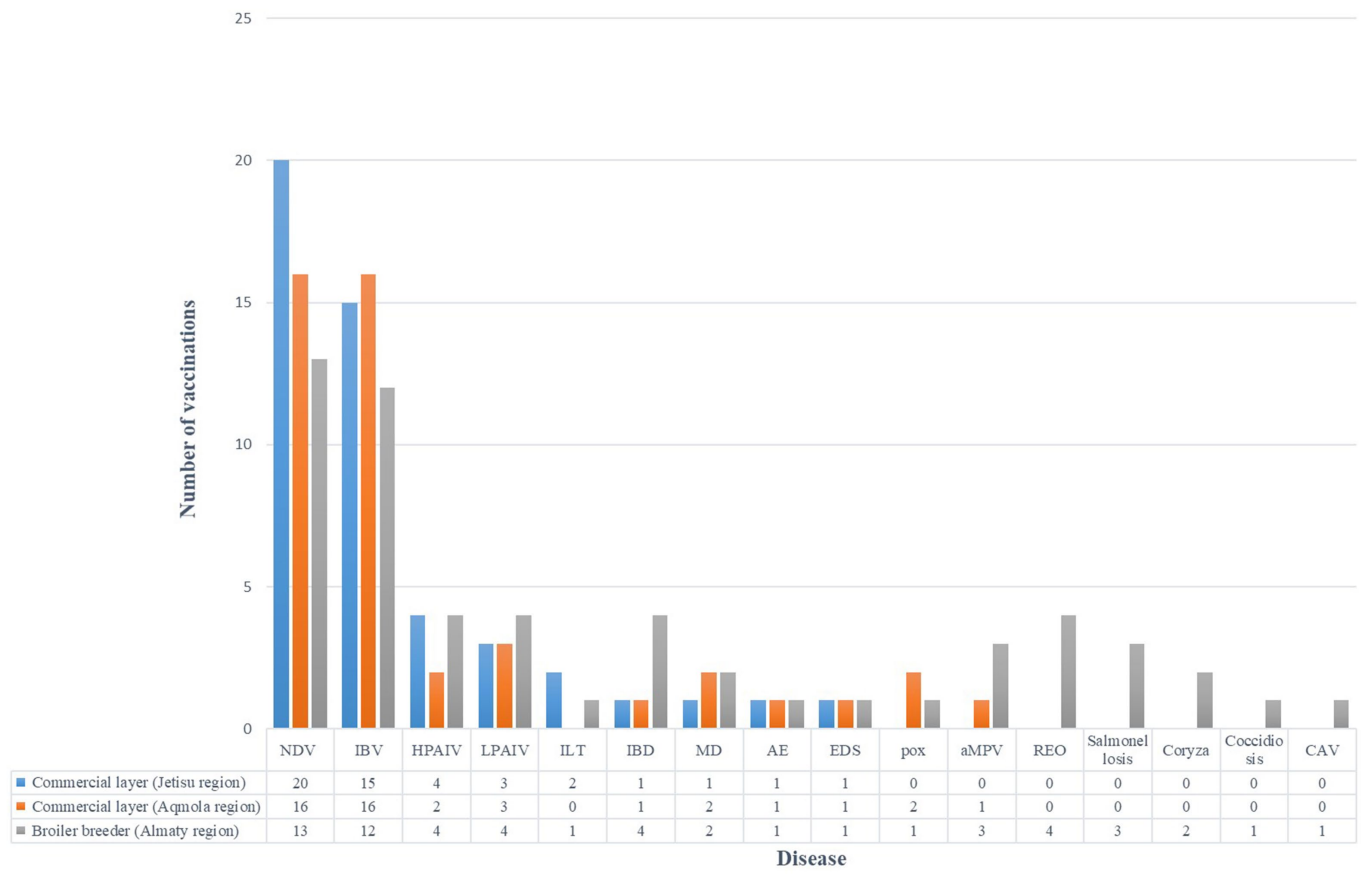
Vaccination frequencies by disease and production type.

The findings suggest a consistent approach to vaccination across the farms in Kazakhstan, with a majority of farms vaccinating against common diseases like ND, IB, HPAIV and LPAIV. Variations in vaccination frequencies were noted for some diseases, such as infectious laryngotracheitis (ILT) and Marek’s disease (MD), but these were not statistically significant across the farm types (Figure 11).

#### 3.6.1 Farm management practices and biosecurity measures

Kazakhstan’s poultry industry spans a diverse range of farm sizes and management practices. Large-scale farms, typically organized as limited liability partnerships (LLPs), follow structured management systems with specialized departments for production and veterinary services. However, these farms face persistent challenges in maintaining adequate staffing levels, particularly for skilled labor, prompting them to recruit workers from neighboring countries.

Smaller, privately operated farms, often run as individual entrepreneurs (IEs), rely on less formal management structures. Both large and small farms implement basic biosecurity measures, including vehicle disinfection, personnel hygiene facilities, and the separation of clean and dirty zones. Despite these efforts, significant challenges remain. Insufficient sanitary downtimes between flock cycles and overcrowding in production facilities—frequently housing mixed-age flocks—hinder effective disease control. Additionally, some farms rely on third-party feed suppliers, which introduces potential risks of feed contamination.

Broilers in Kazakhstan are predominantly raised in floor-rearing systems, while laying hens are kept in cages, and parent flocks are raised as floor broilers. Free-range poultry farming is uncommon in the country. A notable issue is the small number of parent flock farms (8 facilities), which necessitates broiler farms to source hatching eggs from abroad. This reliance on imported eggs increases production costs and biosecurity risks due to potential pathogen introduction.

Disease control strategies in Kazakhstan’s poultry industry reflect a mix of national regulations and international standards. The existing national veterinary regulations, while providing a framework for disease prevention, are outdated and fail to address the complexities of modern poultry farming. To bridge this gap, many large farms adopt internationally recognized management guides, such as those provided by breeding companies, which offer detailed protocols on vaccination, biosecurity, and flock management.

However, the limited involvement of state veterinary services in the poultry sector shifts the burden of disease control to individual farms. This has led to a reliance on farm-specific strategies, which often include the regular use of antibiotics for both treatment and prevention. These practices underscore the need for updated, locally tailored regulations and improved support for disease prevention and management across all farm types.

## 4 Discussion

This study presents an in-depth analysis of the epidemiological dynamics of major avian infectious diseases in Kazakhstan, focusing on HPAI, NDV, MS, MG, IBDV, CAV. Utilizing data from the WOAH, historical literature, and self-conducted laboratory research, this study highlights key findings and their implications for disease control and prevention.

### 4.1 Prevalence and variability of HPAI and NDV

The WOAH plays a crucial role in reviewing setting standards, and providing tools for managing poultry diseases globally. Since 2002, WOAH has focused on HPAI to improve reporting and trade regulations (40). Our study confirms this focus, revealing that HPAI (H5) has a notably higher overall prevalence (10.67%) compared to NDV (4.88%). HPAI prevalence varies widely, from 0.22 to 100%, highlighting the severity of some outbreaks. This variation may be due to differences in biosecurity, bird density, and other factors. In some instances, the observation of 100% prevalence highlights the critical need for regular monitoring and stringent control measures to mitigate the risk of severe outbreaks.

### 4.2 Contextualizing disease prevalence

Our literature review further contextualized the prevalence of poultry diseases within Kazakhstan, providing insights into specific outbreaks and the genetic characterization of pathogens. Studies conducted between 1998 and 2015 highlighted the recurrent nature of Newcastle disease outbreaks across different regions of Kazakhstan, with virulent strains such as genotype VIIb implicated in several instances (16, 17). Additionally, our analysis identified the presence of other pathogens such as aMPV, IBV, and IBDV, underscoring the diversity of infectious agents impacting poultry health in the region (18–21).

### 4.3 Genetic characterization and emerging strains

Through PCR and sequencing, we have identified strains of IBDV. The very virulent strains detected include those identical to MB DQ927040, BG, PLATEAU40/NG/2012 KP152287, and 213–048-2 KY556581.1, among others (34, 35). Another group of very virulent strains identified shared similarities (95,7%) with ISR/2050/2019 MW316418.1, MB DQ927040, BG, KS DQ927042, and PLATEAU40/NG/2012 KP152287, among others (37, 38). Emerging ten years ago in Europe, very virulent strains of the disease have spread swiftly across the globe, causing substantial losses on the poultry sector. Even after a decade, this variant remains a significant concern for the industry (56). Additionally, we identified the SHG308 isolate as an immunosuppressive Chinese variant. American and Chinese IBDV variants exhibited substantial antigenic differences. Sequence analysis of the VP1 and VP2 proteins confirmed this variation, with Chinese strains showing less than 97.7% (VP1) and 98.7% (VP2) sequence identity compared to American variants (36). Furthermore, novel pathogenic viruses in Asia, such as the nVarIBDV strains FJ2019-01 and SHG19 are also significantly different from previously known American IBDV variants (42).

In addition to IBD, we have obtained data on the presence of Newcastle disease in the Akmola and Almaty regions. We isolated genotype VII (subtype VIIi), which is identical to strains such as Waterfowl/1/Istanbul/TR/2018 (MK210599.1), Backyard_chicken/1/Istanbul/TR/2018 (MK210601.1), and PHL159057 (MH371070.1) (39). Additionally, we identified genotype VII.2 (40) and other genotype VII isolates, which are identical to strains like chicken/Iran/SMV-8/2013 (KU201415.1), chicken/Iran/CI1/2017 (MK659696), chicken/Iran/SMV-2/2011 (KU201409), Isf16 (KY205741), and chicken/Iran/CR5/2017 (MK659694) (41). The data obtained indicate that genotype VII, including its various subtypes, is dominant in the control of NDV in Kazakhstan. This genotype was found in different regions and farms where regular vaccination programs against NDV are conducted. Additionally, their nucleotide sequences differ from each other by about 20.53%.

### 4.4 Serologic studies

Furthermore, serologic studies have revealed the seroprevalence of pathogens such as MG and MS, highlighting their distribution and impact on poultry health. An ELISA served as the diagnostic tool for avian mycoplasmosis in this study, which is effective for this purpose (43, 44). The poultry farm with positive samples at the slaughter age for MG and MS was not vaccinated against these diseases, indicating the presence of infection. Additionally, day-old chick samples that tested positive for MG and MS originated from broiler breeders located in Uzbekistan. In contrast, serum samples from Turkey showed no antibodies against these pathogens. Measuring antibody titers in chicks younger than 3–4 weeks is not recommended due to the presence of maternal antibodies, as they have not yet developed their own active immune response (45). However, the vaccination program for broiler breeders from Uzbekistan does not include vaccines against *Mycoplasma synoviae*. Thus, the infection was likely transmitted vertically, which is known as the primary transmission route for MS (46). RT-PCR improves the detection limits for MG infection in chicken breeder flocks. However, studies like Kahya et al. (47) highlight a high match rate (91.4%) between RT-PCR and ELISA, suggesting that ELISA is a reliable diagnostic tool and can serve as an alternative or complementary method to RT-PCR.

The serological analysis for CAV showed a high prevalence of antibodies in both pullets and breeders after vaccination. The average antibody titers ranged from 6,558 to 16,184, exceeding the expected range of 3,000 to 8,000 as outlined in the Biochek results manual. This suggests that the vaccination program effectively induced a strong immune response. However, the wide range of antibody titers, especially the upper values, might indicate variations in the immune response among different flocks or potential over-vaccination. The higher titers in breeders (mean ± SD: 11728.33 ± 2248.04) compared to pullets (mean ± SD: 8371.29 ± 6215.3) could reflect differences in age, immune status, or environmental factors influencing vaccine uptake. The regression analysis results provide additional insights into these variations. The coefficient for suspect titer infection was highly significant (*p* < 0.0001), suggesting that the presence of potential disease significantly correlates with higher antibody levels. This finding emphasizes the need to monitor disease outbreaks in the flock, as these can lead to elevated antibody titers, which may also affect the interpretation of vaccination efficacy. The inadequate vaccination variable also had a significant impact (*p* = 0.045), albeit to a lesser degree, indicating that improper vaccination practices, whether related to dose, timing, or technique, can negatively affect the immune response. This suggests that standardizing vaccination protocols may help reduce the variability in antibody titers observed across flocks. ELISA is recognized as a valuable tool for the routine detection of CAV antibodies in chicken serum. This approach benefits both individual animal testing and broader epidemiological investigations of the virus. However, the presence of CAV antibodies in serum samples requires cautious interpretation. It may indicate past exposure to the virus, either horizontally (through direct bird-to-bird contact) or vertically (from parent to offspring via the yolk sac). Additionally, maternal antibodies, passively transferred through the yolk, can contribute to a positive ELISA result in young birds (48).

The presence of ORT antibodies in non-vaccinated flocks is of significant concern. The study found that 46.67% of pullets and 100% of breeders tested positive for ORT antibodies, with significantly high mean titers (pullets: 9096 ± 8299.52, breeders: 8855.22 ± 4614.54). In addition, the mean titer for suspect infections was significantly higher (15098.76) compared to acceptable titers (5026.14). A *p*-value of <0.0001 strongly supports that this difference is statistically significant, ruling out the possibility of random variation. These findings indicate a widespread exposure to ORT, likely due to environmental transmission, which is well-established occurring through direct contact between birds, inhalation of aerosols, or ingestion of contaminated drinking water. The potential for vertical transmission is still under investigation, although it is considered a possibility (49). The high seroprevalence in breeders further underscores the need for monitoring and potentially introducing vaccination protocols for ORT to mitigate the spread and impact of this pathogen. Vaccination remains the primary strategy for controlling ORT infections, particularly given the widespread antibiotic resistance among the bacteria. However, current vaccines, including autogenous options, have shown mixed efficacy, and economic losses from ORT in developed countries reach hundreds of millions annually. This highlights the need for further research to develop more effective ORT vaccines (50). Therapeutic treatment of ORT infections remains challenging due to widespread antibiotic resistance within the genus. Consequently, prevention is critical and relies on implementing strict hygiene measures, controlling environmental factors and concurrent respiratory pathogens, enforcing thorough disinfection protocols, and combining these efforts with vaccination (49).

The seroprevalence of low pathogenic avian influenza (LPAI) H9 in broiler chickens from the Akmola region revealed a high prevalence of antibodies, with 91.9% of samples testing positive. The mean log2 antibody titer was 3.75 ± 0.712, indicating significant exposure to the virus. Since none of the poultry houses surveyed implemented vaccination against LPAI H9, these findings strongly suggest natural infection and highlight the endemic presence of the virus in the region. The consistent seropositivity observed across different poultry houses and age groups underscores the need for enhanced biosecurity measures and the consideration of vaccination as a control strategy.

Vaccinating commercial broilers with the LPAI H9N2 vaccine has been shown to improve health status, reduce viral shedding, and decrease mortality rates. This protection is particularly effective even under co-infection scenarios involving *Escherichia coli* and LPAI H9N2, mimicking conditions encountered during natural infections (51). Globally, H9N2 viruses are prevalent in wild birds and have become endemic in poultry across various regions of Eurasia and Africa, with ongoing geographic spread. The Eurasian lineage of H9N2 has diversified into three major clades: G1, BJ94/Y280/G9, and Y439/Korean. The lineage evolving in Kazakhstan belongs to the G1 clade (55).

LPAI H9N2 viruses exhibit varying levels of pathogenicity, with H9 alone typically causing limited mortality. However, during respiratory outbreaks, the presence of the virus should be confirmed via PCR. Moreover, the synergistic effects of H9 virus with other pathogens, such as IBDV, MS, avian rhinotracheitis virus (ARTV), and bacteria like ORT, should be carefully evaluated (52).

### 4.5 Survey insights and future directions

A survey conducted between February 29 and March 13, 2024, across 16 poultry farms provided valuable insights into disease incidence and control practices in Kazakhstan. This study highlights significant challenges in disease management within Kazakhstan’s poultry industry. The high prevalence of infectious diseases, such as NDV and bacterial infections (Salmonella spp., *E. coli*, etc.), underscores the need for improved biosecurity and vaccination protocols. These findings align with a previous study analyzing 515 bacterial cultures from poultry across five regions, which identified similar disease patterns (53). Our results provide additional evidence supporting these trends, reinforcing the need for targeted interventions.

Regional analysis revealed distinct disease patterns, with Almaty and Akmola provinces exhibiting similar distributions, while other regions displayed unique trends. Tailored, region-specific strategies are crucial for effective disease control.

Vaccination practices were generally consistent, prioritizing NDV, IBV, and avian influenza. However, gaps remain, such as low vaccination rates against mycoplasmosis. Most farms utilize diagnostic tools like ELISA and HI, though advanced methods such as PCR are less widespread, indicating a need to strengthen diagnostic capabilities (54).

The industry faces additional challenges, including reliance on imported hatching eggs and varying management practices. Poultry farms struggle with biosecurity, overcrowding, and insufficient downtime between flock cycles. Updated national veterinary regulations and increased support for smaller farms are critical to addressing these issues.

In summary, improving biosecurity, expanding vaccination coverage, enhancing diagnostic tools, and updating regulations are essential steps toward mitigating disease impacts and ensuring a sustainable poultry industry in Kazakhstan.

### 4.6 Limitations of the study

While this study provides valuable insights into the epidemiology of poultry diseases in Kazakhstan, several limitations must be considered.

First, the diagnostic methods employed, including PCR and ELISA, may have varying sensitivity and specificity. Variability in diagnostic accuracy across different laboratories, both within and outside Kazakhstan, could influence the reliability of results. Factors such as reagent quality, sample handling, and technical expertise may have introduced inconsistencies, particularly in detecting low-level infections or differentiating between closely related strains.

Second, the survey was limited to a subset of regions, which may not fully represent the disease landscape across the entire country. Farms from only 7 of Kazakhstan’s 17 regions participated, which may limit the generalizability of the findings. Despite distributing the survey to 32 farms, only 16 responded, resulting in a 50% response rate. This limitation likely stems from human factors, including time constraints, limited internet access in certain regions, and lack of interest or availability among farm personnel.

Third, while the study highlights vaccination protocols and biosecurity measures, these data were self-reported by farms and may not fully reflect actual practices. For instance, reported vaccination frequencies and preferences for specific suppliers might not account for unrecorded changes or discrepancies in field implementation. Additionally, although most farms implement biosecurity measures, challenges such as insufficient sanitary downtime between flock cycles and overcrowding in production facilities likely impact disease control efficacy. These practices were not comprehensively evaluated in this study, leaving potential gaps in understanding their true effectiveness.

Lastly, seasonal variations in disease prevalence and environmental factors, such as climate and geographic conditions, were not fully accounted for. These factors can significantly influence pathogen spread and the efficacy of biosecurity measures, potentially biasing the interpretation of disease patterns. Future studies should address these limitations by incorporating a larger and more diverse sample of farms, standardizing diagnostic protocols, and evaluating the on-ground implementation of vaccination and biosecurity strategies.

### 4.7 Future research directions

Future research should focus on tracking the progression of infectious diseases in poultry and evaluating the long-term effectiveness of vaccination and biosecurity measures. Comparative studies across regions can reveal differences in disease dynamics and help tailor prevention strategies. Research on optimizing vaccination protocols, including strain selection and timing, is critical for controlling diseases like NDV, infectious bronchitis virus IBV, and avian influenza.

Molecular studies using genomic sequencing and phylogenetic analysis are needed to better understand pathogen evolution, especially for understudied diseases like IBV and FAdV. Improving diagnostic tools, such as integrating PCR and next-generation sequencing, will enhance early and accurate disease detection.

Exploring the links between environmental, management, and genetic factors and their role in disease prevalence can help address vulnerabilities like overcrowding and reliance on imported hatching eggs. Collaboration between researchers, veterinarians, and poultry farmers is essential for strengthening disease prevention and developing tailored solutions for the industry.

## 5 Conclusion

This study provides critical insights into the epidemiological dynamics and genetic diversity of major avian infectious diseases in Kazakhstan. The findings underscore the importance of continuous surveillance, effective vaccination, and stringent biosecurity measures to control outbreaks and prevent disease spread. A structured biosecurity framework addressing pathogen entry, spread, release, human contamination, and environmental risks is vital for reducing disease impact and protecting public health. Vaccination strategies should target the most prevalent and diverse pathogen strains, incorporating inactivated, live attenuated, and outbreak-specific autogenous vaccines. Future research on the genetic diversity of understudied pathogens and the development of advanced diagnostic tools will further enhance poultry health management and support the sustainability of the industry.

## Data Availability

The data supporting the findings of this study have been deposited in the GenBank repository and are publicly accessible under the following accession numbers: PQ867573, PQ867574, PQ867575, PQ867576, and PQ867577.

## References

[1] Swayne DE Glisson JR McDougald LR Nolan LK Suarez DL Nair VL eds. Diseases of poultry. 13th ed. Wiley-Blackwell: Hoboken, NJ (2013).

[2] OECD/FAO. OECD-FAO agricultural outlook 2021–2030 OECD Publishing (2021). Paris: OECD,

[3] Belova A Smutka L Rosochatecká E. World chicken meat market—its development and current status. Acta Univ Agric Silvicult Mendelianae Brunensis. (2012) 60:15–30. doi: 10.11118/actaun201260040015, PMID: 19514900

[4] Abidov A Mahmudov N Jaxongirov I. Dynamics of development of livestock farming in Uzbekistan and the EAEU countries. E3S Web Conf. (2024) 538:03803016. doi: 10.1051/e3sconf/202453803016, PMID: 38736333

[5] Raha S. Poultry industry in Bangladesh: Present status and future potential. Mymensingh: Agricultural University of Mymensingh (2000).

[6] Rahman MA Rahman MM Abdullah MS Sayeed MA Rashid MH Mahmud R. Epidemiological assessment of clinical poultry cases through the government veterinary hospital-based passive surveillance system in Bangladesh: a case study. Trop Anim Health Prod. (2019) 51:967–75. doi: 10.1007/s11250-018-1782-5, PMID: 30565184

[7] Woo PC Lau SK Yip CC Huang Y Tsoi HW Chan KH. Comparative analysis of 22 coronavirus HKU1 genomes reveals a novel genotype and evidence of natural recombination in coronavirus HKU1. J Virol. (2006) 80:7136–45. doi: 10.1128/JVI.00509-06, PMID: 16809319 PMC1489027

[8] Jones RC. Viral respiratory diseases (ILT, aMPV infections, IB): are they ever under control? Br Poult Sci. (2010) 51:1–11. doi: 10.1080/00071660903541378, PMID: 20390564 PMC7154308

[9] World Bank. World livestock disease atlas: A quantitative analysis of global animal health data (2006–2009). Washington: LSUivestock Unit (2011).

[10] World Organisation for Animal Health (WOAH). Terrestrial animal health code [chapter 4.18. Vaccination, article 4.18.1]. (2023). Available at: https://www.woah.org/en/produit/oie-terrestrial-animal-health-code-2021/

[11] Sandikli MS. Newcastle disease control: the vaccine makes the difference. Int Poultry Prod. (2022) 29:13–5.

[12] World Organisation for Animal Health. World animal health information system—event management. Available at: https://wahis.woah.org/#/event-management [Accessed 12 Sep 2024].

[13] Nuraliev ER. Identifying the causes of bird diseases arising from light disturbance in industrial poultry farming. Vet Zootech Vest Altai State Agrar Univ. (2019) 3:619–36.

[14] Gabbassova Z Dossanova A Lim SS. Current problems and prospects of poultry industry: innovative vector. Probl AgriMarket. (2024) 1:113–24. doi: 10.46666/2024-1.2708-9991.10

[15] Bureau of National Statistics of the Agency for Strategic Planning and Reforms of the Republic of Kazakhstan. Official site of the Bureau of National Statistics of the Agency on Strategic Planning and Reforms of the Republic of Kazakhstan. [Electronic resource]. Date of reference: 13 Feb 2024. Available at: www.stat.gov.kz [Accessed 13 Feb 2024].

[16] Bogoyavlenskiy A Berezin V Prilipov A Usachev E Lyapina O Korotetskiy I. Newcastle disease outbreaks in Kazakhstan and Kyrgyzstan during 1998, 2000, 2001, 2003, 2004, and 2005 were caused by viruses of the genotypes VIIb and VIId. Virus Genes. (2009) 39:94–101. doi: 10.1007/s11262-009-0370-1, PMID: 19466536

[17] Orynbayev M Sultankulova K Kerimbaev AA Strochkov V Shalgynbaev EK Omarova Z. Molecular and biological properties of pathogenic Newcastle disease viruses isolated in Kazakhstan. Sel'skokhozyaistvennaya Biologiya. (2016) 51:255–63. doi: 10.15389/agrobiology.2016.2.255rus

[18] Mussoyev A Assanov N Mussina G Sansyzbai A Valdovska A. Serological aspects of avian metapneumovirus infection in Kazakhstan. Res Rural Dev. (2013):1.

[19] Scherbakova L Ovchinnikova Y Zybina T Kolosov S Zinyakov N Nikonova Z. Infectious bursal disease virus: identification of the novel genetic group and reassortant viruses. Vet Sci Today. (2022) 11:77–84. doi: 10.29326/2304-196X-2022-11-1-77-84

[20] Chechet O Kornienko L Ukhovskyi V Tsarenko T Bilyk S Dovgal V. Potential role of intensive bird growing during outbreaks of viral zoonosis in Ukraine, Russian Federation, Kazakhstan, and Belarus (on the model viruses highly pathogenic influenza and Newcastle diseases): systematic review. J Pure Appl Microbiol. (2022) 16:2363–400. doi: 10.22207/JPAM.16.4.69

[21] Ovchinnikova EV Bochkov YA Shcherbakova LO Nikonova ZB Zinyakov NG Elatkin NP. Molecular characterization of infectious bronchitis virus isolates from Russia and neighbouring countries: identification of intertypic recombination in the S1 gene. Avian Pathol. (2011) 40:507–14. doi: 10.1080/03079457.2011.605782, PMID: 21854179

[22] Pryde P. Environmental resources and constraints in the former soviet republics. 1st ed Routledge (1995). New York.

[23] Tolipov F. Central Asia is a region of five Stans dispute with Kazakh Eurasianists. Central Asia and the Caucasus. (2006) 2:17–26.

[24] Government of the Republic of Kazakhstan, Joint Stock Company “National Managing Holding ‘Baiterek’”. [Electronic resource]. Date of reference: 14 June 2024. Available at: https://baiterek.gov.kz/ru/pr/news/sostoyalsya-zapusk-makinskoy-ptitsefabriki [Accessed 14 June 2024].

[25] Ministry of Agriculture of the Republic of Kazakhstan. [Electronic resource]. Date of address: 14 June 2024. Available at: https://www.gov.kz/memleket/entities/moa/press/news/details/687851?lang=ru [Accessed 14 June 2024].

[26] Association of Legal and Physical Persons “Union of poultry farmers of Kazakhstan”. [Electronic resource]. Date of address: 14 June 2024. Available at: http://ptica.kz/partners

[27] Jozwiak M Wyrostek K Domanska K Olszewska M Smietanka K Minta Z. Application of FTA^®^ cards for detection and storage of avian influenza virus. Bull Vet Inst Pulawy. (2016) 60:1–6. doi: 10.1515/jvetres-2016-0001, PMID: 39726976

[28] Men AE Wilson P Siemering K Forrest S. Sanger DNA sequencing. Next Gen Genome Sequenc 1–11. doi: 10.1002/9783527625130.ch1, PMID: 39723700

[29] Sievers F Wilm A Dineen DG Gibson TJ Karplus K Li W. Fast, scalable generation of high-quality protein multiple sequence alignments using Clustal omega. Mol Syst Biol. (2011) 7:539. doi: 10.1038/msb.2011.75, PMID: 21988835 PMC3261699

[30] Stecher G Tamura K Kumar S. Molecular evolutionary genetics analysis (MEGA) for macOS. Mol Biol Evol. (2020) 37:1237–9. doi: 10.1093/molbev/msz312, PMID: 31904846 PMC7086165

[31] Karamendin K Kydyrmanov A Zhumatov K Asanova S Ishmukhametova N Sayatov M. Phylogenetic analysis of avian influenza viruses of H11 subtype isolated in Kazakhstan. Virus Genes. (2011) 43:46–54. doi: 10.1007/s11262-011-0603-y, PMID: 21461588

[32] Mamadaliyev SM Koshemetov ZK Matveyeva VM Kydyrvayev ZK Zaitsev VL Khairullin BM. Avian influenza virus H5N1 subtype a diagnosed in sick and dead wild and domestic birds in Pavlodar oblast, Republic of Kazakhstan. Afr J Agric Res. (2007) 2:360–5.

[33] Amirgazin A Shevtsov A Karibayev T Berdikulov M Kozhakhmetova T Syzdykova L. Highly pathogenic avian influenza virus of the a/H5N8 subtype, clade 2.3.4.4b, caused outbreaks in Kazakhstan in 2020. PeerJ. (2022) 10:e13038. doi: 10.7717/peerj.13038, PMID: 35256921 PMC8898005

[34] Vera F Craig MI Olivera V Rojas F König G Pereda A. Molecular characterization of infectious bursal disease virus (IBDV) isolated in Argentina indicates a regional lineage. Arch Virol. (2015) 160:1909–21. doi: 10.1007/s00705-015-2449-4, PMID: 26026955

[35] Nwagbo IO Shittu I Nwosuh CI Ezeifeka GO Odibo FJ Michel LO. Molecular characterization of field infectious bursal disease virus isolates from Nigeria. Vet World. (2016) 9:1420–8. doi: 10.14202/vetworld.2016.1420-1428, PMID: 28096615 PMC5234057

[36] Fan L Wu T Hussain A Gao Y Zeng X Wang Y. Novel variant strains of infectious bursal disease virus isolated in China. Vet Microbiol. (2019) 230:212–20. doi: 10.1016/j.vetmic.2019.01.023, PMID: 30827390

[37] Fayyaz M Khan MS Joyia F Zia M. Sequence and structural analysis of synthetic VP2 antigenic protein as a subunit vaccine candidate against very virulent strains of infectious bursal disease virus. Pak. Vet J. (2019) 39:106–10. doi: 10.29261/pakvetj/2018.100, PMID: 39695432

[38] Setta A Yehia N Shaheen M Shami A Al-Saeed FA Alsamghan A. Continuous clinicopathological and molecular recognition of very virulent infectious bursal disease virus in commercial broiler chickens. Poult Sci. (2024) 103:103306. doi: 10.1016/j.psj.2023.103306, PMID: 38228049 PMC10823078

[39] Turan N Ozsemir C Yilmaz A Cizmecigil UY Aydin O Bamac OE. Identification of Newcastle disease virus subgenotype VII.2 in wild birds in Turkey. BMC Vet Res. (2020) 16:277. doi: 10.1186/s12917-020-02503-3, PMID: 32771001 PMC7414739

[40] Reddy N Patil K Shah N Rathod P Chavda N Ruparel F. Deciphering whole genome sequence of a Newcastle disease virus genotype VII.2 isolate from a commercial poultry farm in India. Gene Rep. (2024) 34:101884. doi: 10.1016/j.genrep.2024.101884

[41] Seifi S Khosravi M. Circulation of recently reported sub-genotype VII1.1 of Newcastle disease virus in commercial and backyard chicken in north of Iran. Journal Name (2021);1.

[42] Wang C Hou B. The booster immunization using commercial vaccines effectively protects chickens against novel variants of infectious bursal disease virus (genotype A2dB1). Poult Sci. (2024) 103:103552. doi: 10.1016/j.psj.2024.103552, PMID: 38422756 PMC10910156

[43] Cadman HF Kelly PJ Zhou R Davelaar F Mason PR. A serosurvey using enzyme-linked immunosorbent assay for antibodies against poultry pathogens in ostriches (*Struthio camelus*) from Zimbabwe. Avian Dis. (1994) 38:621–5. doi: 10.2307/1592088, PMID: 7832718

[44] Abdelmoumen BB Roy RS. An enzyme-linked immunosorbent assay for detection of avian mycoplasmas in culture. Avian Dis. (1995) 39:85–93. doi: 10.2307/1591986 7794195

[45] Camba SI Rovira HG. Comparison of serologic methods for the detection of *Mycoplasma gallisepticum* (MG) antibodies in broilers in South Luzon, Philippines. Philipp J Vet Anim Sci. (2023) 49, 33–42.

[46] ter Veen C de Wit JJ Feberwee A. Relative contribution of vertical, within-farm and between-farm transmission of Mycoplasma synoviae in layer pullet flocks. Avian Pathol. (2020) 49:56–61. doi: 10.1080/03079457.2019.1664725, PMID: 31509002

[47] Kahya S Temelli S Eyigor A Carli KT. Real-time PCR, culture, and serology for the diagnosis of *Mycoplasma gallisepticum* in chicken breeder flocks. Vet Microbiol. (2010) 144:319–24. doi: 10.1016/j.vetmic.2010.01.012, PMID: 20149561

[48] Gholami-Ahangaran M. Serological and molecular detection of chicken anaemia virus in Iranian poultry flocks. Vet Ital. (2015) 51:211–5. doi: 10.12834/VetIt.80.253.3 26455374

[49] Barbosa EV Cardoso CV Silva RCCF Cerqueira AMF Liberal MHT Castro HC. *Ornithobacterium rhinotracheale*: an update review about an emerging poultry pathogen. Vet Sci. (2020) 7:3. doi: 10.3390/vetsci7010003, PMID: 31892160 PMC7157751

[50] Gornatti Churria CD Vigo G Machuca M Nievas V Nievas W Piscopo M. Vaccines against *Ornithobacterium rhinotracheale*: a review. J Vet Sci Med Diagn. (2013) 2:1–4. doi: 10.4172/2325-9590.1000122

[51] Mahmoud SIA Zyan KA Hamoud MM Khalifa E Dardir S Khalifa R. Effect of co-infection of low pathogenic avian influenza H9N2 virus and avian pathogenic *E. coli* on H9N2-vaccinated commercial broiler chickens. Front Vet Sci. (2022) 9:918440. doi: 10.3389/fvets.2022.918440, PMID: 35836502 PMC9274096

[52] Youk SS Lee DH Jeong JH Pantin-Jackwood MJ Song CS Swayne DE. Live bird markets as evolutionary epicentres of H9N2 low pathogenicity avian influenza viruses in Korea. Emerg Microbes Infect. (2020) 9:616–27. doi: 10.1080/22221751.2020.1738903, PMID: 32183621 PMC7144223

[53] Biyashev KB Makbuz AZ Biyashev BK. Occurrence of enteroinfectious pathogens in agricultural animals and poultry. Biol Med. (2016) 8

[54] Liebhart D Bilic I Grafl B Hess C Hess M. Diagnosing infectious diseases in poultry requires a holistic approach: a review. Poultry. (2023) 2:252–80. doi: 10.3390/poultry2020020

[55] Thiermann AB. The new world organisation for animal health standards on avian influenza and international trade. Avian Dis. (2007) 51:338–9. doi: 10.1637/7671-062606R.1, PMID: 17494578

[56] van den Berg TP. Acute infectious bursal disease in poultry: a review. Avian Pathol. (2000) 29:175–94. doi: 10.1080/03079450050045431 19184804

